# Herpes simplex virus spreads rapidly in human foreskin, partly driven by chemokine-induced redistribution of Nectin-1 on keratinocytes

**DOI:** 10.1371/journal.ppat.1012267

**Published:** 2024-06-10

**Authors:** Hafsa Rana, Naomi R. Truong, Blake Johnson, Heeva Baharlou, Jason J. Herbert, Sasikaran Kandasamy, Robert Goddard, Ralph C. Cohen, Michael Wines, Najla Nasr, Andrew N. Harman, Kirstie M. Bertram, Kerrie J. Sandgren, Anthony L. Cunningham

**Affiliations:** 1 The Westmead Institute for Medical Research, Westmead, New South Wales, Australia; 2 Faculty of Medicine and Health, The University of Sydney, Sydney, New South Wales, Australia; 3 Research and Development, Vaxxas Pty Ltd., Brisbane, Queensland, Australia; 4 University of Sydney and Australian National University, Children’s Hospital at Westmead, New South Wales, Australia; 5 Urology, Sydney Adventist Hospital, Wahroonga, New South Wales, Australia; Washington State University, UNITED STATES

## Abstract

HSV infects keratinocytes in the epidermis of skin via nectin-1. We established a human foreskin explant infection model to investigate HSV entry and spread. HSV1 entry could only be achieved by the topical application of virus via high density microarray projections (HD-MAPs) to the epidermis, which penetrated beyond one third of its thickness, simulating *in vivo* microtrauma. Rapid lateral spread of HSV1 to a mean of 13 keratinocytes wide occurred after 24 hours and free virus particles were observed between keratinocytes, consistent with an intercellular route of spread. Nectin-1 staining was markedly decreased in foci of infection in the epidermis and in the human keratinocyte HaCaT cell line. Nectin-1 was redistributed, at the protein level, in adjacent uninfected cells surrounding infection, inducible by CCL3, IL-8 (or CXCL8), and possibly CXCL10 and IL-6, thus facilitating spread. These findings provide the first insights into HSV1 entry and spread in human inner foreskin *in situ*.

## Author summary

Herpes Simplex Virus (HSV) infects 3.7 billion people globally, leading, in some, to lifelong recurrent disease and co-infection with viruses such as HIV. There is no cure or vaccine. Animal models and *in vitro* cell lines do not accurately represent the initial events that take place during HSV infection in human genital mucosa. We have successfully established a model of acute HSV-1 infection within explants of human inner foreskin, a common site for sexual transmission. Topical microtrauma penetrating a third of the way into the epidermis was essential for HSV infection, indicating that the two most superficial strata were refractory. There was rapid spread of HSV1 particles through and around epidermal keratinocytes over 24 hours. HSV1 particles also interacted with and were taken up by epidermal Langerhans Cells (LCs) and Dendritic Cells (Epi DCs). Focal HSV1 infection of keratinocytes induced a redistribution of nectin-1, the HSV entry receptor, in a collar surrounding the foci via specific chemokines which further facilitated viral spread. These results provide an insight into the initial events that lead to HSV1 infection and spread within human genital mucosa and defines a time window of opportunity to target the virus before it enters epidermal nerves and becomes latent.

## Introduction

Herpes simplex viruses (HSV) types 1 and 2 cause genital herpes. HSV2 is the most common cause of recurrent herpes, but HSV1 is an increasingly common cause of initial genital herpes and predominant in some populations [1]. As shown in biopsies of primary and recurrent lesions [2,3], both viruses target the keratinocytes in the stratified squamous epithelium that makes up the epidermis of skin, in addition to two types of mononuclear phagocytes (MNPs): Langerhans cells (LCs) and dendritic cells (DCs). Viral spread is restricted to the epidermis. This epidermis consists of the deep *stratum basale* and successively superficial layers of the *stratum spinosum*, *stratum granulosum* and *stratum corneum*, with the upper two layers providing barrier protection from pathogens. Thus, a breach or inflammation of the epithelium in skin appears to be required to facilitate viral penetration [4,5]. Sexual intercourse, with either anal or vaginal penetration has been shown to induce microabrasions and trauma to anogenital mucosa [6,7]. In separate studies, 55% of women who engage in consensual sex and up to 73% of both women and men have been shown to undergo some type of anogenital injury [8,9]. Studies in Kenya also showed 64–66% of men self-reported penile injuries during consensual sex, with a higher risk of injury in uncircumcised men [10,11].

Studying the mechanisms and kinetics of HSV entry and spread within the epidermis of inner foreskin will elucidate 1) The time window prior to viral entry into the sensory nerve endings within the epidermis and subsequent transport to the dorsal root ganglia (DRG) where it establishes lifelong latency and 2) The kinetics of initiation of the immune response via MNPs. Rapid infection and spread in keratinocytes, to the ‘sanctuary’ of terminal axons of sensory neurons provides HSV with an efficient strategy to evade the immune response [12,13]. Thus, understanding these events could guide strategies for vaccines or immunotherapy to prevent viral spread to the nerves and establishment of a lifelong infection.

Uptake of HSV1/2 by epidermal LCs and DCs is essential for initiation of the immune response to HSV infection. We recently showed that the two MNPs, LCs and DCs are infected by HSV1 via different routes [14]. LCs internalised HSV1 via langerin-mediated, pH-dependent endocytosis and became productively infected which triggered apoptosis. LCs migrated into the dermis while apoptosing and clustered with dermal type 1 conventional DCs (cDC1s) [3], which have a propensity to cross-present viral antigens to CD8^+^ T cells. Epidermal DCs (Epi DCs) internalised HSV1 via pH-independent pathways, yet still apoptosed due to productive viral infection similar to LCs [14]. Epidermal MNPs can directly sample HSV as it penetrates the skin, or via infected keratinocytes.

HSV infects keratinocytes via receptors nectin-1 and, to a minor degree, Herpesvirus Entry Mediator (HVEM), as studied in human HaCaT and nTERT keratinocyte cell monolayers or differentiated multilayers and in murine skin explants [4,15]. After initial infection of HaCaT or nTERT keratinocytes, progeny virus infects neighbouring cells via direct cell-cell spread, requiring HSV glycoproteins gD, gE and gI, or via interstitial spaces, but can be nectin-independent [16].

Keratinocytes have three major cell junction protein complexes that play different roles in adhesion and epidermal barrier formation; tight junctions, adherens junctions, and desmosomes. In skin, all cell-cell junction proteins are located in close proximity to each other within the keratinocyte membrane to regulate and maintain this barrier [17,18], with tight junctions being most superficial in nongenital skin. Nectin-1, the major entry receptor for HSV, also functions as an adherens junction protein to maintain the epidermal barrier and regulate the exchange of fluids and other molecules, as well as in the formation of desmosomes [19,20]. Desmosomes are complex multi-protein structures, including the adaptor protein, Plakoglobin, which is also found in adherens junctions [20]. Inflammation results from the depletion of Ca^2+^ and disruption of Ca^2+^-dependent tight junctions in skin [21,22]. This causes a breakdown in epidermal integrity and redistribution of nectin-1 from the adherens junction to the entire cell surface which leads to increase binding of HSV1 gD [23]. In human and murine skin explant studies, mechanical trauma also led to viral infection around the wounded area, due to tight junction disruption, but did not spread to cells with intact tight junctions [4,5,24,25]. However the epithelial distribution of these three intercellular junctions in cervico-vaginal mucosa and in inner foreskin differs markedly from abdominal or murine trunk skin, likely to reduce the barrier function of tight junctions in the upper epidermis and permit luminal exchange [26,27].

Here we introduced three substantial innovations to more closely simulate the events of genital herpes and to more accurately track viral spread. Firstly, we developed a HSV infection model using human inner foreskin explants, a clinically relevant site of infection. Inner foreskin is a type II mucosal tissue with a thin *stratum corneum* and contains an abundance of cytokine secreting immune cells making it more vulnerable to pathogens such as HIV [28–30]. Secondly, we studied the role of microtrauma in infection by introducing HSV into the tissue using High-Density Microarray Patches (HD-MAPs). Thirdly, in addition to using GFP-labelled virus, we utilized a more sensitive detection system–RNAscope *in situ* hybridization (ISH), to detect HSV1 DNA. LCs and Epi DCs interacted with HSV within foci of infection but were reduced in density. HSV only infected keratinocytes when microneedles punctured at least a third of the depth of the epidermis, below the *stratum granulosum*, and the virus spread rapidly within 24 hours suggesting an intercellular route in addition to cell to cell spread. HSV infection caused redistribution of nectin-1 *in situ*, with reduced staining in infected cells. In HSV1^+^ HaCaT cell cultures, there was a redistribution of nectin-1 in the uninfected cells surrounding HSV foci, via keratinocyte-secreted chemokines, especially CCL3, CXCL10, IL-6 and IL-8, thus facilitating viral spread.

## Results

### Development of an HSV-infected inner foreskin model to investigate the initial events of HSV infection and spread

In preliminary experiments, RNAscope was used to detect intracellular HSV1 DNA, specifically the long unique region (UL) -30, and individual HSV1 particles after topical application of HSV1 to explants of child and adult inner foreskin. Explants were infected with HSV1 via a cloning cylinder attached to the surface of the epidermis of the inner foreskin and cultured for 24 hours. HSV1 was detected but only on the mucosal surface, without any keratinocyte infection (**S1A Fig**). Therefore, without a breach in the epidermis, especially of the *stratum corneum*, HSV was not able to enter the skin of children or adults.

**Fig 1:**
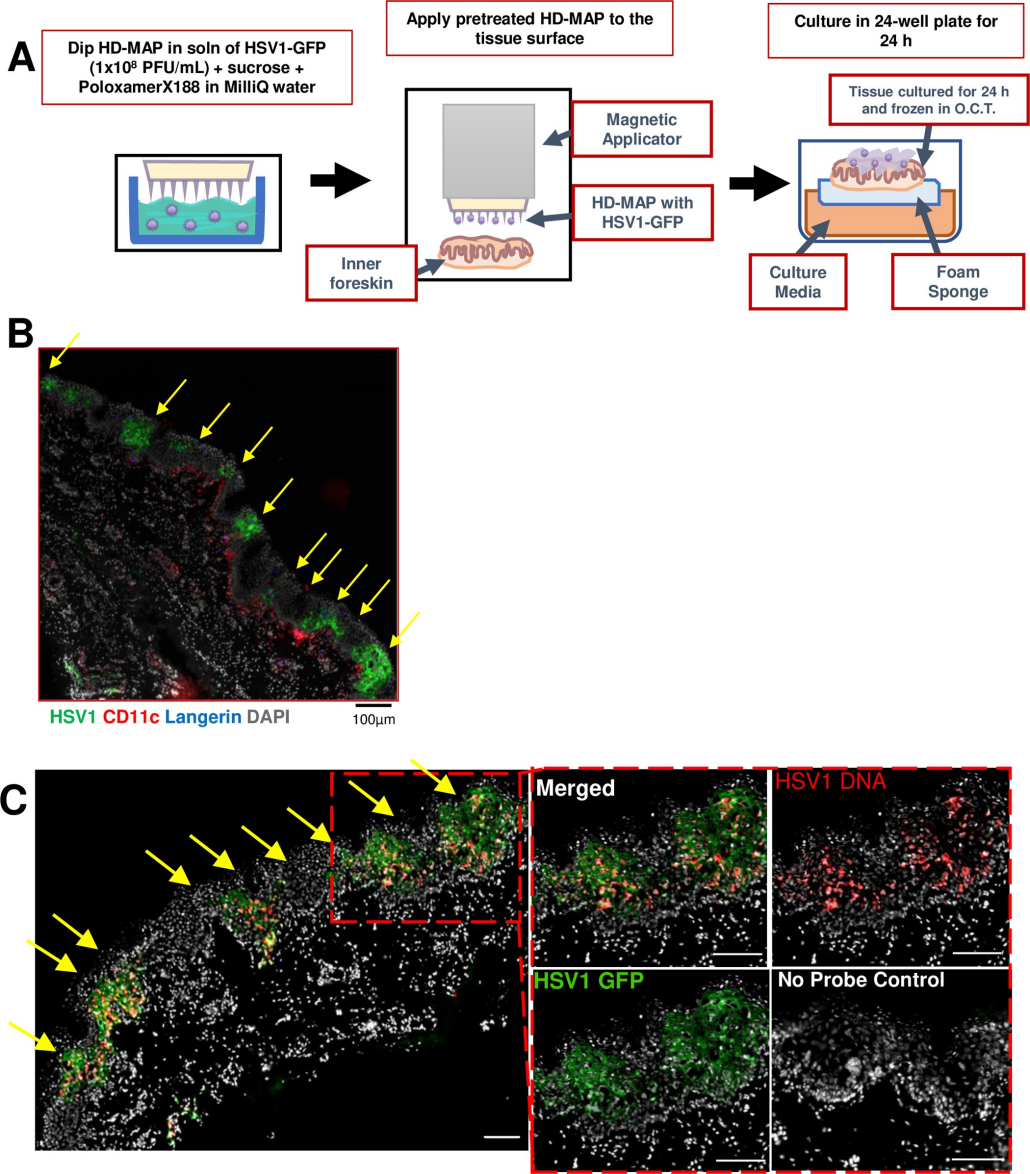
Development of an explant infection model to investigate the initial events during HSV epidermal infection. **(A)** Vaxxas HD-MAPs were pre-treated with HSV1-GFP (1x10^8^ PFU/mL) and then applied to inner foreskin tissue via a magnetic applicator. Tissue was cultured for 24 hours at 37°C and snap-frozen in OCT. **(B-C)** Cryosectioned 24-hour HSV1-GFP infected inner foreskin was labelled with **(B)** rabbit anti-human GFP primary and donkey anti-rabbit Alexa Fluor (AF)-488 secondary (green), mouse anti-human CD11c primary and donkey anti-mouse AF647 (red) and goat anti-human Langerin primary and donkey anti-goat AF755 secondary (blue) antibodies and DAPI nuclear stain (grey). **(C)** Anti-GFP (green), HSV1 DNA via RNAscope (red) and DAPI nuclear stain (grey). HSV1 infection spreads outwards from punctures as indicated by the yellow arrows. Scale bars = 100 μm.

As microtrauma is known to facilitate viral entry into human skin, HD-MAPs were used to induce microtrauma in inner foreskin epidermis (**Fig 1A**). These HD-MAPs were applied using an automatic mechanical applicator and induced punctures in adult epidermis to a depth of 40±20 μm or approximately 40% or less of the epidermal thickness (**S2 Fig**), thus inducing consistent punctures that did not penetrate beyond the basement membrane (BM) into the dermis, allowing efficient epidermal viral entry. Analysis of multiple child foreskin samples (ages 5–14 y.o.) and adults (ages 17 y.o. and above) showed that the thickness of the epidermis increases with age (**S2A and S2B Fig**). The child inner foreskins had a thinner epidermis of 100±3 μm in comparison to adult inner foreskins which had a thickness of 150±25 μm. Infant foreskins (under age 5) with even thinner epidermis were omitted from this study as most (80±5%) punctures penetrated the BM (**S2B Fig**) and could facilitate atypical infection within the dermis. After patch-treatment, topical application of the virus did not result in viral entry into the epidermis. Thus, the HD-MAPs were coated with a solution containing HSV1-GFP (tagged to US9, a late infection protein) before application to the tissue (**Fig 1A**). Intra-epidermal infection of human inner foreskin was detected by GFP signal at 24 hours post infection (h.p.i.) in association with microneedle punctures (**Fig 1B**). The virus infected the keratinocytes surrounding the punctured regions as indicated by the yellow arrows, forming viral plaques within the epidermis, similar to herpetic lesions.

**Fig 2:**
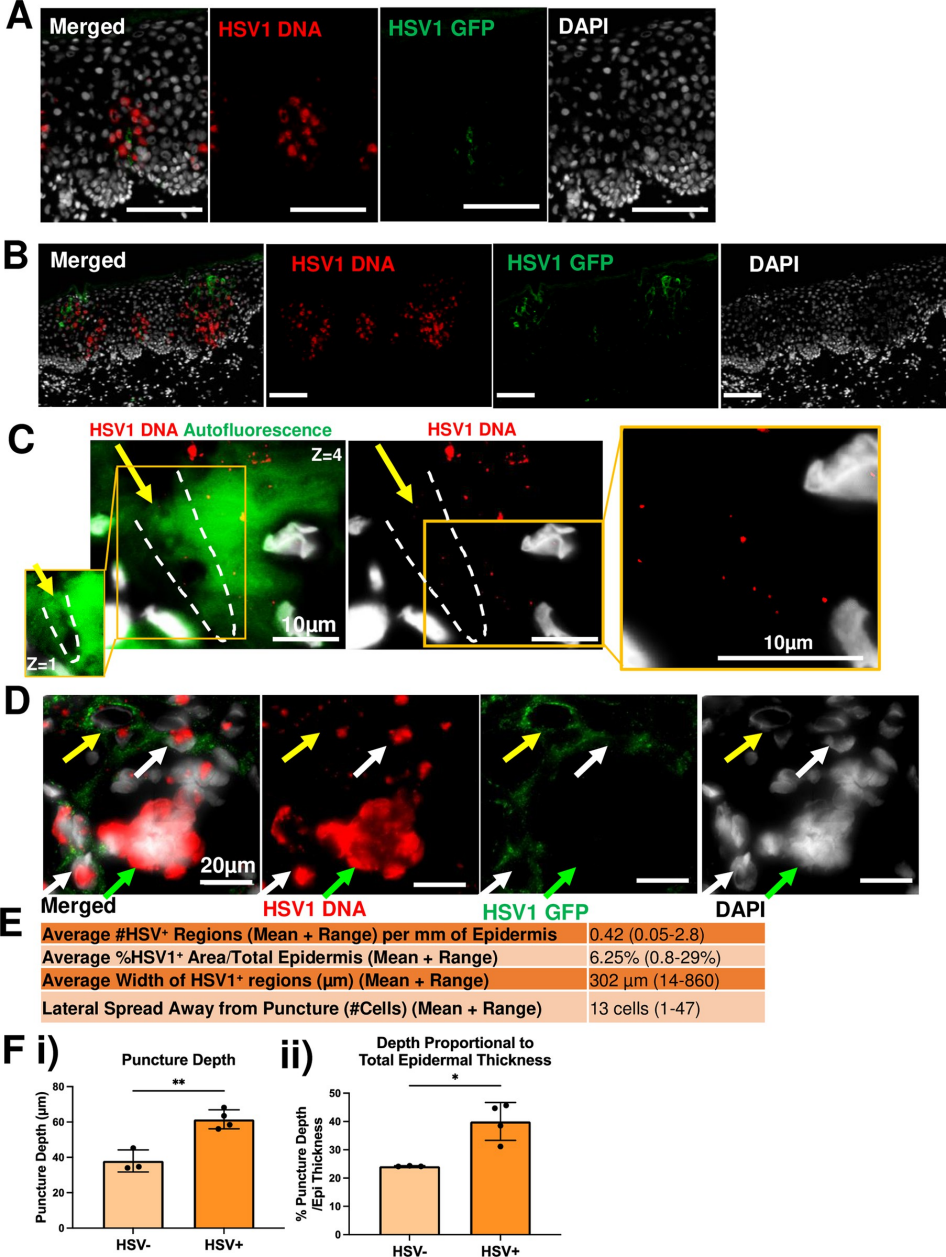
Intraepithelial microneedle penetration of greater than 30% of the epidermis is required to establish detectable intra-epidermal infection in anogenital tissue. **(A-D)** Cryosectioned 24-hour HSV1-GFP infected inner foreskin was labelled by RNAscope to detect HSV1 DNA (red), anti-GFP antibody (green) and DAPI nuclear stain (grey). Scale bars = 100 μm unless indicated otherwise. **(A)** HSV1 infected keratinocytes of the mid-epidermis, but not upper layers, as indicated by HSV1 DNA. **(B)** HSV1 infection cascade is shown with HSV1 DNA only detected in lower epidermis, and both DNA and GFP detected in upper layers. **(C)** Single virions or small aggregates of particles (right inset: zoomed in image of smaller particles) of HSV1 DNA observed within the puncture region (dotted line; left inset: zoomed in image in different Z plane) indicated by the yellow arrow at 100x magnification, imaged using the Olympus VS200 Slidescanner. **(D)** Nuclei of infected cells show various infectious states within keratinocytes; nuclear globular DNA alone (green arrow), nuclear globular DNA with cytoplasmic GFP (white arrows), single punctate virions and cytoplasmic GFP (yellow arrow). **(E)** A table of averages for the measurements of all HSV1^+^ explants infected with HSV1 including; the average number of HSV1^+^ regions per mm of epidermis, proportion of HSV1^+^ area, and average size of the HSV1^+^ foci in width (μm) and in number of cells (n = 5). **(F)** 24-hour HSV1-GFP infected inner foreskin samples were classified as HSV^-^ (no expression of GFP or DNA) or HSV^+^ (expressing GFP and/or DNA) (i) the puncture depth and (ii) the puncture depth proportional to the total epidermal thickness were measured. Data presented as mean ± S.D. (HSV^-^: n = 3, HSV^+^: n = 4). * = p<0.05, determined by unpaired parametric t tests with Welch’s correction assuming unequal variances.

HSV1 DNA was also targeted using RNAscope and showed a large quantity of viral DNA being produced by infected keratinocytes (**Fig 1C**). Using both RNAscope and GFP-labelling, which labelled a late stage protein, facilitated the detection of different stages of infection within the explants, as HSV1 DNA in foci was detected in some samples that did not express any GFP signal and also in others at the periphery of GFP^+^ foci, indicating that those keratinocytes were at an earlier stage of infection (**Table 1 and Fig 2A and 2B**). Occasionally, individual virions or small viral aggregates were seen within the epidermis, most clearly within the punctures (indicated by yellow arrow and dotted line), but also between keratinocytes (**Fig 2C**). DNase pretreatment of HSV1-infected tissue showed that the presence of these virions or aggregates was not due to free DNA from the viral inoculum (**S3 Fig**). HSV1 DNA was detected within infected cells and often in cells co-expressing GFP, indicating late viral protein production (**Fig 2D**). HSV1 DNA was mostly detected within the nuclei of infected keratinocytes where it appeared focal in some cells and diffuse in others, and in some cells also within the cytoplasm, in the presence or absence of GFP. Analysis of six HSV1^+^ foreskin samples showed that at 24 h.p.i. there was a mean of 0.42 (with a range of 0.05–2.8) HSV1^+^ regions per mm of epidermis (**Fig 2E**). The average HSV1^+^ area made up 6.25% (with a range of 0.8–29%) of the total area of epidermis and the average width of an infected region was 326 (with a range of 14–860) μm. There was great variability in lateral spread of the infection away from the puncture within 24 hours, with an average spread of 13 cells from the puncture in two dimensions, and a range of 1–47 cells within one section. As the HD-MAPs were 1cm x 1cm with a density of 10 000 microneedles, one row contains 100 microneedles and with an average of 5.46 microneedle punctures per mm of epidermis. This lead to approximately 8% of microneedles inducing HSV1 foci. Where foci were small these were usually located within the *stratum spinosum* of the mid-epidermis, indicating infection could be initiated in this region. Larger foci were spread across almost all layers of the epidermis and spread laterally as well. Thus, microtrauma facilitated the establishment of large regions of intra-epidermal HSV1 infection in human inner foreskin tissue with substantial lateral viral spread within 24 hours.

**Table 1:** Summary of all genital tissue samples treated with HSV1-GFP via pre-treatment of high-density microarray patches*.

Sample	Age	Inflammation status	HSV1-GFP+/-	HSV1 DNA+/-
**1**	5	Mild inflammation	+++	+++
**2**	43	Healthy	-	-
**3**	12	Healthy	+	+
**4**	41	High inflammation in lower lamina propria	++	++
**5**	14	Healthy	-	-
**6**	17	Healthy	-	-
**7**	18	Healthy	-	-
**8**	9	Healthy	+++	+++
**9**	21	Mild inflammation	-	++
**10**	22	High inflammation	-	+
**11**	11	Healthy	-	-
**12**	11	High inflammation	-	-
**13**	35	Healthy	-	-

*Excluding controls

- = No infection as indicated by lack of DNA or GFP detection

+ = low infection as indicated by DNA or GFP detection

++ = mild infection as indicated by DNA or GFP detection

+++ = high infection as indicated by DNA or GFP detection

Of the 13 samples processed, approximately half became infected, as assessed by HSV1 DNA and/or HSV1-GFP expression (**Table 1**). Further analyses of the puncture wounds were performed to determine if the punctures had affected the level of virus penetration into the epidermis in infected and non-infected samples (**Fig 2F**). The HSV^+^ samples all showed a deeper puncture depth (60±5 μm) than the HSV^-^ samples (38±5 μm) which corresponded to 40±5% and 25±1% of total epidermal thickness in these samples respectively. These results indicate that the microneedles needed to penetrate at least 30–35% of the epidermal thickness, and pass the most superficial layers of keratinocytes (the *strata corneum* and *granulosum*), to establish detectable intra-epidermal infection.

### Langerhans Cells and Epidermal DCs interact with HSV1-infected keratinocytes and become infected

**Fig 3:**
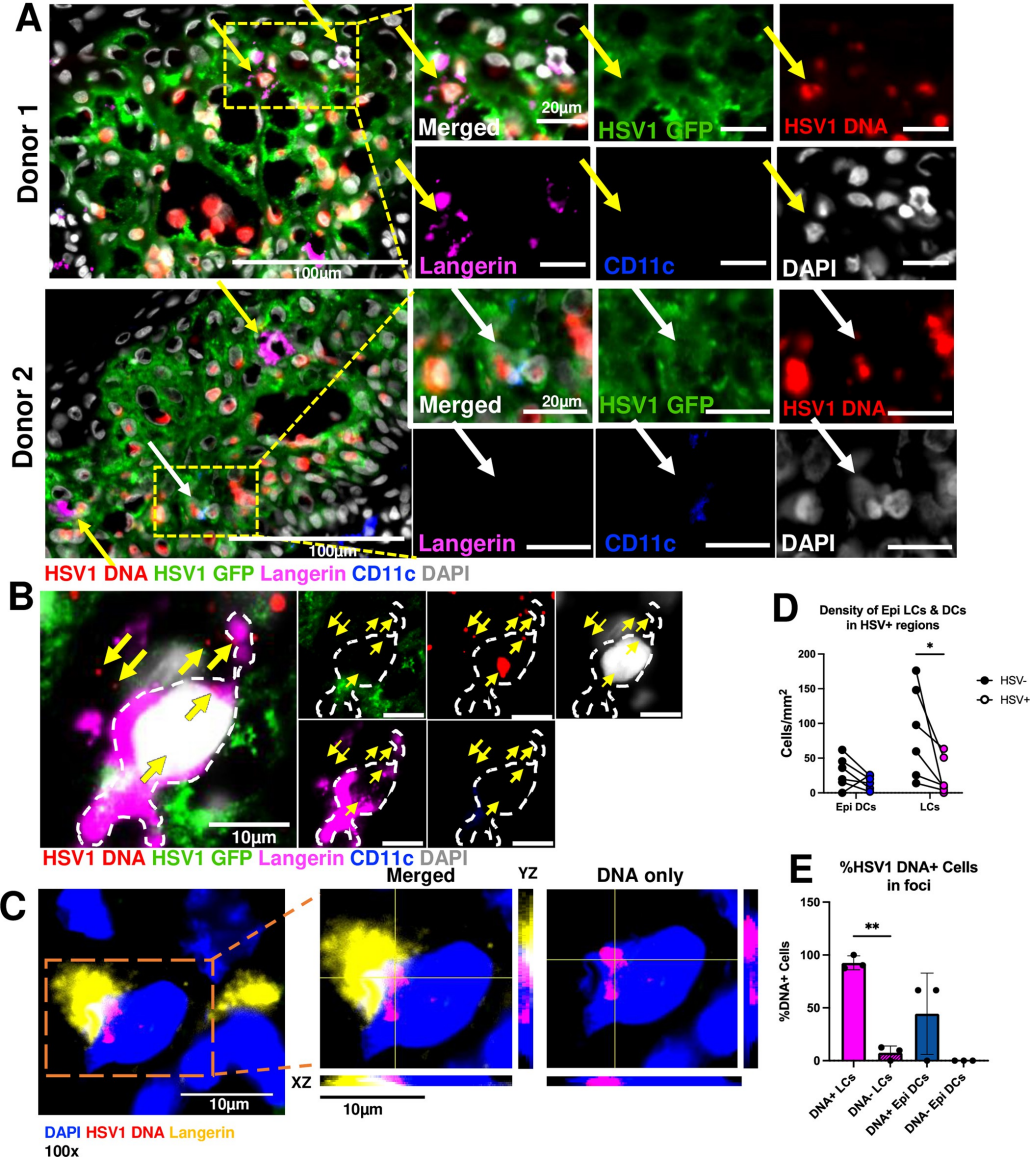
Epidermal MNP subsets interact with HSV1-infected keratinocytes and become productively infected. **(A and B)** Cryosectioned 24-hour HSV1-GFP infected inner foreskin from two samples labelled by RNAscope to detect HSV1 DNA (red), anti-GFP (green), anti-CD11c (blue) and anti-Langerin (magenta) antibodies and DAPI nuclear stain (grey). **(A)** Yellow arrows indicate HSV1 DNA^+^ LCs, white arrows indicate HSV1 DNA^+^ Epi DCs. Scale bars = 20 μm unless indicated otherwise. **(B)** Yellow arrows indicate HSV1 DNA surrounding a langerin^+^CD11c^-^ LC and localised to the nucleus. Scale bars = 10 μm. **(C)** High resolution Z-stack with orthogonal view inset showing the colocalisation of DAPI (blue) and HSV1 DNA (red) overlapping to appear magenta, in a langerin^+^ (yellow) LC. Image acquired on Olympus VS200 Slide Scanner at 100x magnification. Scale bars = 10 μm. **(D)** Pair-wise comparisons of the mean densities of LCs in HSV^-^ (black circles) and HSV^+^ (magenta circles) regions, and Epi DCs in HSV^-^ (black circles) and HSV^+^ (blue circles) regions. * = p<0.05, determined by paired t tests with Wilcoxon’s matched pairs signed rank test. **(E)** Percentage of HSV DAPI^+^DNA^+^ and DAPI^+^DNA^-^ Epi DCs of total DCs within foci presented as mean ± S.D. (n = 3). * = p<0.05, determined by paired parametric t tests.

LCs (CD11c^-^langerin^+^) and Epi DCs (CD11c^+^langerin^+/-^) interacted with HSV1 and HSV1-infected keratinocytes in multiple samples, indicated by yellow and white arrows respectively (**Fig 3A**). Epidermal LCs and DCs were observed in the infected regions as well as the surrounding areas, and as shown by RNAscope, and LCs were observed engaging in uptake of and infection by HSV1 (**Fig 3B and 3C**). High resolution Z-stacking confirmed the colocalization of HSV1 DNA within the nucleus of LCs, demonstrating these cells were infected (**Fig 3C**). Previous studies have shown LCs emigrate out of the tissue in response to external stimuli including viral infection [31]. We observed that there was no significant decrease in the density of Epidermal LCs and DCs over 24h in untreated tissue, suggesting that only a minority of cells appeared to migrate out of the tissue after patch treatment (**S4A and S4B Fig**). Thus, there was no significant further overall cell loss after treatment with patches, although a decreased density around punctures was noted (**S4C Fig**). Overall, there was very little change in the distribution of the epidermal LCs and DCs over time with or without patch-treatment, indicating that these cells remain dispersed in the epidermal tissue and are still prime targets for viral infection, even in the presence of microtrauma. However, both subsets showed a decrease in cell density within HSV1-infected regions with a significant decrease in LCs (**Fig 3D**). Quantification of the number of HSV1 DNA^+^DAPI^+^ cells showed 90±5% of LCs within infected foci contained HSV1 DNA, as opposed to 45±40% of Epi DCs (**Fig 3E**).

### Productive HSV1 infection of keratinocytes leads to decreased nectin-1 staining in epidermal explants

**Fig 4:**
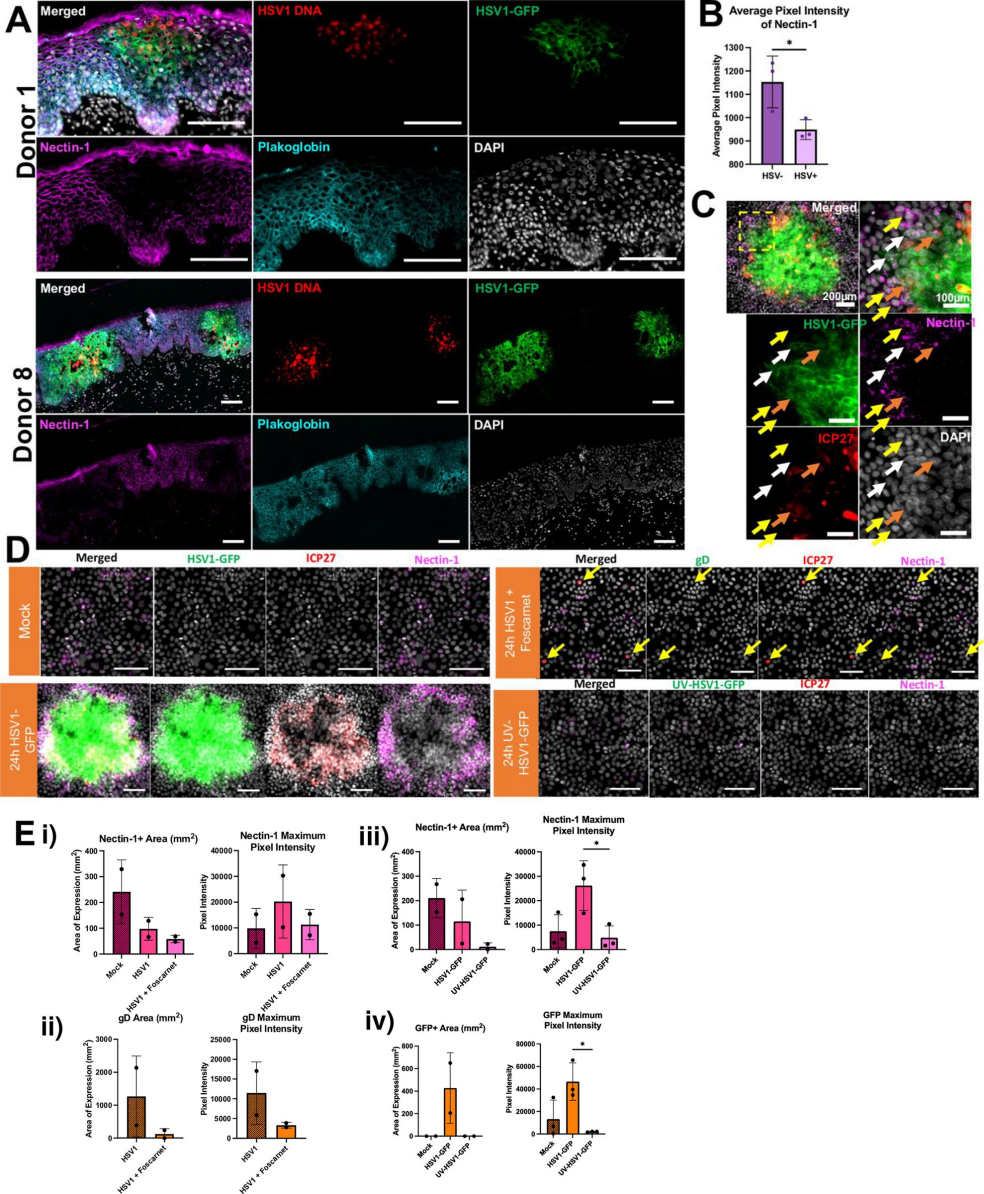
Nectin-1 staining is decreased in HSV1-infected keratinocytes, but enhanced as a collar in surrounding uninfected cells. **(A)** Cryosectioned 24-hour HSV1-GFP infected inner foreskin from two samples labelled by RNAscope to detect HSV1 DNA (red), anti-GFP (green), mouse anti-human Nectin-1 primary conjugated in house to Cyanine5 (Lumiprobe) and mouse anti-human Plakoglobin (PG, cyan) primary followed by donkey anti-mouse AF755 secondary antibodies and DAPI nuclear stain (grey). Scale bars = 100 μm. **(B)** Pixel intensity of nectin-1 within HSV1-GFP^+^ or negative epidermal regions within the same tissue presented as mean ± standard deviation (n = 3). * = p<0.05, determined by an unpaired t test. **(C)** 24h HSV1-GFP infected HaCaT cells with arrows indicating ICP27^+^GFP^+^Nectin-1^+^ (orange), ICP27^-^GFP^+^Nectin-1^+^ (white) and ICP27^-^GFP^-^Nectin-1^+^ (magenta) cells. Scale bars = 100 μm unless stated otherwise. **(D)** HaCaT cells cultured for 24 hours with either serum-free medium only (mock), HSV1 or HSV1-GFP (MOI 0.1), pre-treated with Foscarnet (1 mg/mL) for 1 hour (and remaining in the culture thereafter), or treated with UV-inactivated HSV1-GFP (UV-HSV1-GFP). Cells were labelled with anti-gD (green; when treated with untagged HSV1), anti-ICP27 (red) and anti-Nectin-1 (magenta) antibodies and DAPI nuclear stain (grey). Yellow arrows indicate gD^-/low^ICP27^+^Nectin-1^-^ cells. Scale bars = 100 μm. **(E)** The average area (mm^2^) and maximum pixel intensity for (i) nectin-1 and (ii) gD expression in mock, and HSV1 vs HSV1 + Foscarnet treated HaCaT cells, n = 2 and iii) nectin-1 and iv) GFP expression in mock, HSV1-GFP and UV-HSV1-GFP treated HaCaT cells. * = p<0.05 determined by an ordinary one-way ANOVA with Tukey’s multiple comparisons test.

To determine the effects of microtrauma and HSV1 infection on the distribution of nectin-1, we aimed to visualise nectin-1 and its localisation to the intercellular junctions between keratinocytes in inner foreskin epidermis. There are three types of intercellular junctions; tight junctions, adherens junctions, and desmosomes. We used Plakoglobin, which resides within both desmosomes and adherens junctions [20], as a control marker for intercellular junctions, to distinguish specific effects on nectin-1 (contained in adherens junctions) rather than the integrity of all intercellular junctions. RNAscope for HSV1 DNA was performed along with HSV1-GFP detection and anti-nectin-1 and anti-plakoglobin IF labelling in inner foreskin at 24 h.p.i.. Within the HSV1^+^ regions of all infected explants, nectin-1 staining was markedly decreased at 24 hours, where the distinct reticulated pattern marking the interface of the cell membranes observed in uninfected control epidermis, was completely absent on infected cells (**Fig 4A**). However, plakoglobin staining remained unchanged within the keratinocyte junctions, indicating that this phenomenon was specific to nectin-1, although plakoglobin staining did decrease in areas where epithelial integrity was lost. Quantitatively, there was a significant decrease in the staining intensity of nectin-1 in HSV1^+^ regions compared to HSV1^-^ regions (quantified as the average pixel intensity) within the tissue (**Fig 4B**). Nectin-1 has previously been shown to be reduced in HSV1^+^ cell lines [32,33]. Here, for the first time we observed a similar effect on HSV1-infected keratinocytes *in situ* in human foreskin tissue.

### HSV1 infection reduces nectin-1 staining in infected foci and redistributes nectin-1 on adjacent uninfected cells around the foci

We next used HaCaT cells to further investigate the dynamics of nectin-1 localisation which are similar phenotypically to the keratinocytes of the *stratum spinosum*, as determined by their expression of keratins 10 and 14 and function [34,35]. HaCaT cells grown on coverslips were mock or HSV1-GFP-infected for 24 hours (**Fig 4C and 4D**). Within mock-infected HaCaT cells, nectin-1 staining was absent in most cells, and where dim, was usually localised to the cell membranes (**Fig 4D**), presumably due to their location within adherens junctions, which may prevent efficient antibody binding [23]. Foci were first detected by ICP27 and GFP staining between 6 and 12 hours (**S5 Fig**). Nectin-1 staining was bright in some of the infected cells which co-expressed ICP27^+^ and/or GFP^+^ indicative of earlier stage infection (**Fig 4C**). Nectin-1 staining was reduced in foci within 24 hours post infection (with increased expression of HSV1-GFP). Of particular note, the cells immediately surrounding the infected foci (ICP27^-^GFP^-^), showed increased nectin-1 staining. This was confined to the edges of the foci forming a defined collar or rim around them (**Fig 4D**).

To determine whether these changes in nectin-1 staining were due to productive HSV1 infection or a direct interaction with gD on the virus (as shown previously by Barghava et al. [33], HaCaT cells were treated with HSV1-GFP for 24 hours and compared to cells treated with Foscarnet for 1 hour prior to HSV1 infection (**Fig 4D**). Only a few single cells were HSV1^+^ within the Foscarnet treatment, as expected (indicated by yellow arrows and lower gD expression as shown in **Fig 4E**). However there was no early increase in nectin-1 staining in infected cells, nor the signature collar of nectin-1^+^ cells surrounding the infected cells at 24 h.p.i.. This was further proven by quantification of the nectin-1^+^ area (100±50mm^2^) and maximum pixel intensity (20 000±15 000) when treated with HSV1 alone, compared to a decrease of about 50% in both when pre-treated with Foscarnet (**Fig 4E**). UV-inactivated HSV1-GFP was also used to determine whether nectin-1 redistribution could be induced by direct contact and entry of the inactivated virus (via surface gD) with keratinocytes. The pattern of staining of nectin-1 did not follow the serial increase and decrease in expression in HSV1-GFP-infected cells, nor was there any nectin-1 staining in uninfected cells (**Fig 4D and 4E**), indicating that direct interaction of the cell and virus surface glycoproteins (especially gD) is not sufficient to induce changes in nectin-1. Thus, HSV1 replication, late protein production and/or spread is essential for the redistribution of nectin-1 in foci and in surrounding cells.

### HSV1 infected keratinocytes produce inflammatory cytokines and chemokines that may regulate nectin-1 redistribution, in response to HSV1 infection

**Fig 5:**
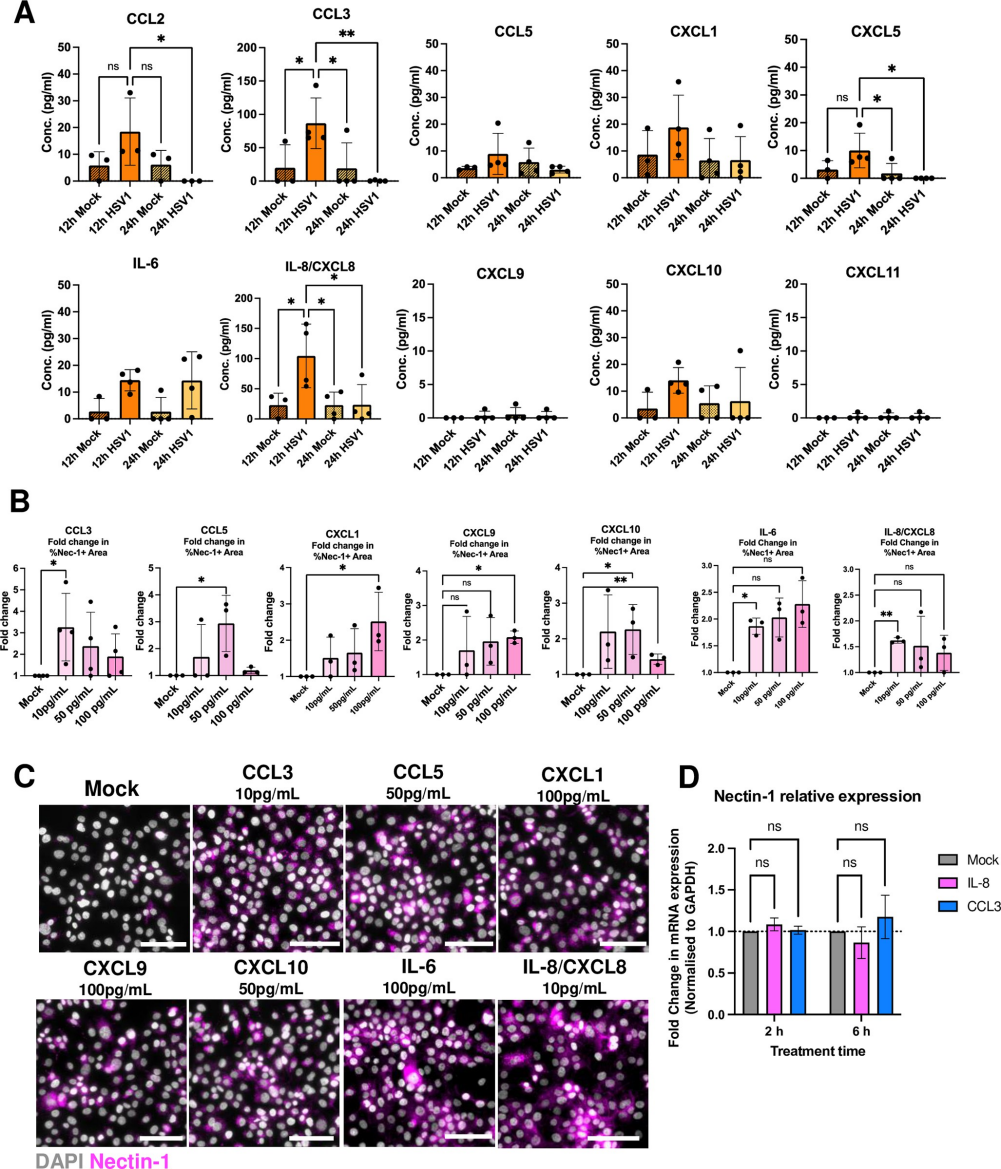
HaCaT keratinocytes produce inflammatory chemokines in response to HSV1 infection, which increase nectin-1 redistribution. **(A)** HaCaT cells cultured for 12 or 24 hours with either serum-free medium (mock) or HSV1-GFP (MOI 1). Supernatants were analysed for an array of cytokines and chemokines via the LEGENDplex Assay (BioLegend). Concentrations (pg/mL) of detected cytokines and chemokines are displayed as mean ± S.D. (n = 4). * = p<0.05 determined by an ordinary one-way ANOVA with Dunnett’s multiple comparisons test. **(B)** HaCaT cells were treated with serum-free media (mock) or CCL3, CCL5, CXCL1, CXCL5, CXCL9, CXCL10, IL-6 and IL-8 at 10, 50 or 100 pg/mL for 6 hours and labelled with anti-Nectin-1 (magenta) antibody and DAPI nuclear stain (grey). Fold change in the percentage of the area that is nectin-1^+^, and relative to the mock, is displayed as mean ± S.D. (CCL3: n = 4, all others: n = 3). * = p<0.05, ** = p<0.01, *** = p<0.001, determined by repeated measures one-way ANOVA with Dunnett’s multiple comparisons or single unpaired parametric t tests. **(C)** Representative images of HaCaT cells treated with the concentrations required for optimal response are displayed. Scale bars = 100 μm. **(D)** HaCaT cells were treated with serum-free media (mock) or CCL3 or IL-8 at 10 pg/mL for 2 and 6 hours. RNA was extracted and RT-qPCR was performed using primers to nectin-1 and GAPDH. The fold change in nectin-1 expression by IL-8 and CCL3 treatment normalised to GAPDH is shown, displayed as mean ± S.D. (n = 2). Statistical analysis to assess data significance was determined by repeated measures two-way ANOVA (Treatment x Time) with Dunnett’s multiple comparisons test.

We reasoned that the collar of increased nectin-1 staining in uninfected HaCaT cells, around HSV1 plaques was likely the result of diffusing soluble mediators, probably chemokines or cytokines. To assess the mechanism behind this paracrine effect, the supernatants of HaCaT cells infected with HSV1-GFP for 12 hours or 24 hours were examined for various inflammatory cytokines and chemokines. HSV1-infected HaCaT cells at 12 h.p.i. produced significantly increased levels of CCL3 and IL-8, with trends in CCL2, CCL5, CXCL1, CXCL5 and CXCL10, all of which decreased by 24 h.p.i., especially for CCL2, CCL3, CXCL5 and IL-8 (**Fig 5A**). Only IL-6 was produced at both 12 and 24 h.p.i. in most samples. Interestingly, CXCL9 and CXCL11 were not detected at either timepoint in HSV1-infected HaCaT cells, although both chemokines, along with CXCL10, are ligands of CXCR3. We observed similar trends in the supernatants of HSV1-GFP infected foreskin explants at 24 h.p.i. (**S6 Fig**).

Treatment of HaCaT cells for 6 hours with CCL3, CCL5, (both targeting CCR1 expressed by keratinocytes), CXCL1, IL-8 (targeting CXCR2), CXCL9 and CXCL10 (targeting CXCR3) and IL-6 (targeting IL-6R) followed by IF labelling of nectin-1 showed a significant increase in the extent of nectin-1 redistribution in the HaCaT cell monolayer at cytokine concentrations of 10 and/or 50–100 pg/mL (**Fig 5B and 5C**). CCL2 and CXCL5 did not significantly increase nectin-1 staining, thus this data was excluded. These studies took into account the cycling of nectin-1 expression in HaCaT cells (minimal at 18 hours post seeding, **S7 Fig**). RT-qPCR was performed to measure changes in nectin-1 expression in HaCaT cells at the transcriptional level in response to IL-8 and CCL3, however no significant change was seen in comparison to mock-treated cells (**Fig 5D**). This strongly suggests that nectin-1 redistribution, not up or downregulation, is occurring at the protein level. Thus, it appears that HaCaT cells respond to the early stages of HSV infection by producing these pro-inflammatory cytokines and chemokines, but this is diminished by 24 hours, probably an effect of HSV-induced inhibition of transcription of cellular RNAs and subsequent cell death. This increase in pro-inflammatory cytokine and chemokine release coincided with the increase in nectin-1 at 12 h.p.i.

## Discussion

This study aimed to develop a model of acute HSV infection at the site of sexual transmission and determine the cellular and cytokinetic factors that enhance viral spread within the epidermis. After topical administration of HSV to explants of human inner foreskin, HSV did not enter the intact epidermis, and microtrauma, via HD-MAPs, was required for entry into this tissue by bypassing the two upper strata of keratinocytes. Entry was probably also aided by initial redistribution of the surface expressed nectin-1 out of sequestration in the adherens junctions on keratinocytes abutting the punctures. Pre-coating of the projections of HD-MAPs with HSV1 prior to application to the tissue was strictly necessary to establish infection. No infection was established if the punctures were introduced prior to adding the virus. This suggests that for transmission, virus containing fluids must be present at the time of microtrauma during intercourse *in vivo*. Infection of inner foreskin explants from multiple samples showed that HSV1 spread laterally away from the puncture at an average of 13 cells in each infected focus within 24 hours. As a single viral replication cycle of HSV1 takes approximately 12–18 hours [36,37], the duration of infection *in situ* represented 1–2 rounds of cell infection at most and yet managed to spread across 13 cells, on average. This suggests that virus is not only spreading cell-to-cell via *de novo* replication, but also through the interstitial spaces or across the surfaces or cell membranes of adjacent keratinocytes without infection. This rapid spread could involve viral ‘surfing’ [38] or partly by other mechanisms.

This study expanded our previous studies that used GFP labelled virus by incorporating RNAscope, enabling the detection of HSV1 DNA in individual particles and small aggregates in punctures, within and between cells for the first time. RNAscope was used in our previous study to detect HIV interactions with epidermal LCs and DCs within inner foreskin epidermis [30]. Using RNAscope for HSV1 DNA we were able to show earlier stages of infection in several different distribution patterns; 1) Focal nuclear DNA detection in infected keratinocytes, presumably representing the earliest accumulation within ND10 domains [39,40]; 2) Diffuse nuclear staining, probably mostly as nuclear capsids; 3) Nuclear and cytoplasmic staining, the latter presumably in capsids and assembling virions; 4) Nuclear DNA and cytoplasmic US9-GFP in infected keratinocytes. Some DNA-expressing groups of keratinocytes were also seen that did not express any HSV1-GFP which may be due to early stages of infection, or that only early infection was established (i.e. abortive infection).

At first glance our results appear to be in contrast to reports from the Knebel-Morsdorf laboratory [4,5] which found that in explants of human abdominal and breast skin and oral mucosa and murine skin, mechanical trauma was insufficient for topical HSV-1 entry and infection. They established successful HSV infection of skin by stripping the dermis and submerging the epidermis in a viral solution, resulting in infection of the basal epithelial layers and spread superficially, but only to the *stratum spinosum* as the *stratum granulosum* did not become infected even when treated with mechanical abrasions. However, this does not appear to explain sexual transmission *in vivo*. Although we could initiate topical infection of genital mucosal explants by automated patch delivery of HSV, we also found that only epidermal layers below the *stratum granulosum* (i.e. below 30–35% of the depth of the epidermis) could be infected. Such infection could be initiated in the *stratum spinosum* as well as the deeper *stratum basale*. Indeed small and larger foci of HSV infection were often confined to the *stratum spinosum* in the mid-epidermis without involving basal layers, The *stratum granulosum* is probably refractory to HSV1 infection due to marked biological changes including increased accumulation of keratin and lipids [5,25,41,42].

Recent results from the same group, using reconstituted epidermis from dissociated human keratinocytes, also suggested that tight junction formation superficial to the adherens junctions containing nectin-1 was a major barrier to HSV interacting with nectin-1 after topical addition. However, conditions simulating atopic dermatitis allowed such infection. There are several reasons for the differences to our system. Firstly, we could achieve topical infection by viral coating of the HD-MAPs. Secondly, spread of HSV from punctures and through interstitial spaces clearly led to expansion of foci of infected keratinocytes consistent with the marked differences in the distribution of intercellular junctions in the upper strata of inner foreskin epithelium resulting in a reduced barrier function, compared to external skin [26,27]. Thirdly, our RNAscope technique is far more sensitive in detecting viral infection, including individual or aggregated virions, than conventional immunofluorescence staining. Together, the results from the two laboratories are consistent with human *in vivo* HSV infection and disease which is common in the genital tract but uncommon in external skin, unless the patient has an underlying inflammatory skin disease, such as atopic dermatitis.

Our study also contributes further understanding of the interaction between HSV and resident LCs/DCs in the epidermis of human genital mucosa after topical infection, adding to our previous work. The Epi DCs and LCs within inner foreskin epidermis, were distributed throughout the epidermis, allowing them to encounter incoming viruses and pathogens. *In situ* quantification showed a similar concentration of both epidermal subsets [30]. We previously showed HSV replicates within LCs to express structural proteins such as GFP-labelled pUL37/US9 in inner foreskin [3], using IF microscopy after topical application to the surface of infant inner foreskin tissue. Unlike RNAscope used here, this technique does not detect particle uptake nor early infection. In the absence of microtrauma, infection of LCs, but only of occasional keratinocytes, were detected. Probably, the thinner upper epidermal strata in infants allowed such LC infection but the sequestration of nectin-1 in adherens junctions in the absence of trauma explained the lack of keratinocyte infection. In this study, within HSV-treated epidermis, the cell density for both LCs and Epi DCs was lower adjacent to the HSV1^+^ regions than to HSV1^-^ regions, suggesting that the infected cells migrate out of the epidermis to interact with dermal cells, as previously shown [3]. In a more recent study, we showed that both infected LC and Epi DCs undergo apoptosis *in vitro* [14] and thus we presume both cell types migrate to the dermis in response to HSV1 infection, while undergoing apoptosis, to be taken up by dermal DCs. This will be addressed in future studies.

A limitation of this foreskin explant model is its ‘closed-system’ nature, being isolated from the vascular system carrying soluble factors, maintaining tissue oxygenation and immigration of circulating inflammatory cells, such as plasmacytoid DCs, monocytes, and T cells which control HSV infection. However, this does not diminish the biological relevance of such a model as it very closely resembles the tissue microenvironment that HSV would initially encounter at early times *in vivo*. We found that the epidermis remains intact during *ex vivo* culture for duration of our experiments (up to 24 hours) as determined by histology. Cellular composition and relationships within the epidermis were also not significantly altered within this time. Another limitation of this model is sample variability in tissue samples, requiring increased number of replicates for significant results.

The role of the HSV1/2 receptor nectin-1 is essential to our understanding of the initial events of HSV infection within genital tissue. It is highly expressed on keratinocytes and LCs/DCs [4,16,30,33,43]. Nectin-1 staining was completely and specifically diminished in HSV1-infected areas within the epidermis, however the normal reticular staining pattern remained intact within the surrounding uninfected cells. Plakoglobin expression was maintained in the foci, suggesting disruption of adherens junctions but not desmosomes between HSV1-infected keratinocytes [5,17,24]. We chose to model the effects of HSV1 infection on nectin-1 redistribution in the human keratinocyte HaCaT cell line as they can express abundant nectin-1 and HSV spread in these cells has been studied by a number of laboratories [44,45]. Although HaCaT cells are an *in vitro* model, they are representative of the suprabasal layers of the epidermis, characterised by their expression of keratins 1 and 10 [46,47] where HSV1 infection was established. There was natural cycling of nectin-1 in HaCaT cell cultures (although not at the transcriptional level), suggesting this cycling was due to nectin-1 protein redistribution between adherens junctions in the cell membrane and the cytoplasm (**S7 Fig**). We quantified HSV1 and chemokine effects at the time of minimal nectin-1 staining (i.e. 18 hours post-seeding), clearly showing a marked reduction in nectin-1 staining within infected foci and its inhibition by Foscarnet. The collar of redistributed nectin-1 in the adjacent cells surrounding the area of infection were ICP27^-^GFP^-^, indicating that they were not yet infected (or not yet expressing viral proteins), but exposed to nectin-1-inducing soluble factors, probably cytokines and chemokines secreted from infected foci. For comparison, changes in nectin-1 also occurred in the majority of HSV-transfected mouse melanoma cells within a monolayer but were induced by relatively few virions, perhaps suggesting that the interaction induces a paracrine effect on surrounding cells [33].

Chemokines from HSV1-infected HaCaT keratinocytes showed a tendency to increase at 12 h.p.i. then decrease again by 24 h.p.i., correlating with the kinetics of nectin-1 redistribution. Our studies in infected HaCaTs and to some degree infected explants expanded our previous data on cytokine and chemokine production by HSV infected keratinocytes and correlates with those in recurrent herpes vesicle fluid [48]. Both studies showed production of IL-6, -10 and -12 and CCL2, -3, and -5 and changes over time in culture (or for Mikloska et al. [48], times of sampling of vesicle fluid). However, our data also shows enhanced production of IL-6, IL-8 and a much wider range of chemokines. Addition of CCL3 and CCL5, which bind to keratinocyte-expressed CCR1; CXCL1, CXCL5 and IL-8 which bind to CXCR2; and CXCL9 and 10, which bind to CXCR3, showed increased staining of nectin-1 by the HaCaT cell monolayer. This was significant for CCL3 and IL-8 with marked trends for the others. CXCL9, 10 and 11 were also shown to play a critical role in HSV infection within mice [49,50]. The individual variability in chemokine effects, may be due to chemokine receptor-targeting by alternate chemokines in different people. The signalling pathways downstream from these chemokine receptors intersect, leading to NFκB activation and suggesting a potential mechanism for nectin-1 redistribution, which will be tested in future experiments [51–55]. IL-6, the ligand for IL-6 receptor (IL-6R) also induced nectin-1 redistribution. However, whether this receptor-ligand interaction activates NFκB has been variable in previous reports [56–60]. Nevertheless, crosstalk between STAT3, PI3K and NFκB pathways has been reported previously and may explain the redistribution of nectin-1 within keratinocytes [61,62]. Therefore, our data shows that keratinocytes are producing cytokines and chemokines that enhance nectin-1 redistribution on neighbouring cells and lead to the redistribution of the entry receptor from the cellular membrane to facilitate viral spread.

In conclusion using a novel combination of HSV DNA detection, an authentic genital mucosal explant and a system for reproducible microtrauma, this study provides insight into events that resemble those occurring *in vivo*. The results strongly suggest that sexual transmission of HSV requires induction of microtrauma penetrating the *stratum spinosum* during sexual intercourse. There was rapid spread of virions probably via intercellular spaces within 24 hours to produce infected foci of keratinocytes on average 13 cells wide and of both types of epidermal MNPs, suggesting prevention or therapy should be aimed at free virus as well as infected cells. In HaCaT culture models, spread was facilitated by chemokine induction of a collar of redistributed nectin-1 in uninfected keratinocytes surrounding the foci of infected cells which secreted the relevant chemokines CCL3 and IL-8, as well as IL-6, CXCL1, CXCL5, CXCL9 and CXCL10. LCs and Epi DCs also take up virus and appear to migrate out of the epidermis, consistent with the pathway published previously on the early steps of the immune response [3]. Further work is underway to confirm that Epi DCs also undergo apoptosis and migrate to the dermis as do LCs.

## Methods

### Ethics

This study was approved by the Western Sydney Local Area Health District Ethics Committee(reference numbers: 4192-2019/ETH01894 & 4192 AU RED HREC/15 WMEAD/11). Written consent was received from all participants or legal guardians of infants or young children donating tissue for the study.

### Materials availability

This study did not generate any new materials or reagents. All other reagents are readily and commercially available internationally.

### Cell lines

HaCaT cells (donated by Dr. Laurence Levy [63]) were cultured in DMEM supplemented with 10% FBS in 75 cm^2^ vented-cap flasks and passaged every 3 days once they reached 80–90% confluency.

### Viruses

HSV1 stocks were prepared using Vero cells grown in 150 cm^2^ flasks in DMEM supplemented with 2% FBS. HSV1 strain F expressing GFP-US9 (HSV1-GFP; donated by Prof. Renato Brandimarti [64] and HSV1 strain F were propagated in Vero cells over two days. Cells were then harvested, and freeze-thawed three times. Sonication was performed using the Branson 450 Cup Digital Sonifier (400 W) three times for 20 seconds each with 70% amplitude and 40 second intervals between pulses to release the cell-associated virus. Cellular debris was pelleted by centrifuging at 4000 g for 10 minutes at 4°C. Supernatants were stored at -80°C. Virus infectivity was determined via plaque assays using Vero cells where plaques were manually counted, and the plaque-forming units (PFU) were calculated. An aliquot of the sonicated HSV1-GFP was inactivated by UV light exposure. The virus was placed under UV light at a minimal distance of ~5 cm in a plate with the lid removed and incubated three times for 20 minutes with intermittent rocking to ensure even and maximum exposure. The virus was then aliquoted and stored at -80°C until use. Plaque assays were performed using Vero cells to ensure inactivation was effective.

### Tissue samples

Paediatric (0–14 y.o.) and adult (17+ y.o.) foreskin tissue was obtained from circumcision surgeries via collaborations with surgeons across the Greater Sydney Region. Tissue samples were acquired within 30 minutes of surgery and were mostly healthy unless stated otherwise by the surgeon. Inflammation status was validated by microscopy as determined by cellular infiltrate (**Table 1**). Outer foreskin was removed from samples via a scalpel and forceps prior to setting up explant infections. Tissues were cryosectioned and stained for histology using a Haematoxylin and Eosin stain.

### Inner foreskin explant infections

Magnetic 1cm^2^ HD-MAPs supplied by Vaxxas (Australia) consisting of 10 000 silicon microprojections 250±20 μm in length were used to simulate microtrauma. Initially, HSV1-GFP applied topically after treatment with HD-MAPs did not prove successful in aiding viral entry, thus the HD-MAPs were coated with virus prior to application to the tissue. The magnetised HD-MAPs were dipped ten to twenty times using forceps in a 1 mL solution of HSV1-GFP (1 x 10^8^ PFU/mL), 15% sucrose (w/v) and 0.05% Poloxamer 188 (Sigma-Aldrich), a detergent used to reduce the surface tension of the patch and enhance coating of the needles [65], diluted in RPMI-1640 within a 12-well plate and then applied to the inner foreskin using a magnetic applicator (Vaxxas, Australia). HD-MAPs without any treatment were applied to the tissue as a mock control. Tissue explants were then excised from surrounding tissue using a scalpel and placed onto GelFoam (Pfizer, USA), pre-soaked in “DC culture medium” (pH 7.5) containing RPMI-1640 (Lonza) supplemented with 10% fetal calf serum (Sigma-Aldrich), 10 μM HEPES buffer, 1 mM sodium pyruvate, 1X non-essential amino acids, 5 μg/mL Gentamicin & 50 μM 2-mercaptoethanol, within a 24-well plate and incubated at 37°C and 5% CO_2_ for 24 hours. Explants were then placed in flat 15 x 15 mm cryomolds (Tissue-Tek) in O.C.T compound and snap-frozen in liquid nitrogen in a 2-methylbutane bath and stored at -80°C. Frozen tissue was sectioned at 7 μm using the HM505 cryostat (Microm Int. GmbH) in preparation for Haematoxylin & Eosin stain, RNAscope and immunofluorescence (IF) microscopy. All images were acquired on the Olympus VS120 Virtual Slide Microscope using the 20x objective (unless stated otherwise) and the Olympus VS-ASW software version 2.9 or the Olympus VS200 Slide Scanner using the 100x objective (unless stated otherwise) and the Olympus VS200 ASW software version 3.4. Image analysis was performed using FIJI (ImageJ) software version 1.53a.

### RNAscope and immunofluorescence staining of foreskin tissue

Detection of HSV1 DNA was performed using the RNAscope 2.5HD Red Reagent Kit (ACD Bio) as per the RNAscope 2.5HD Manual protocol with a readily available probe against HSV1 DNA targeting *UL30* (Cat no. 498861). Briefly, frozen tissue sections were fixed in 2% PFA for 20 minutes at room temperature (RT), treated with BLOXALL (Vector Laboratories, USA) for 10 minutes, followed by a 20-minute Protease Plus treatment (diluted 1:5 in cold PBS) and a 2-hour incubation with the HSV1 DNA probe at 40°C performed in a HybEZ hybridisation oven (220VAC). Probes were validated via DNase I and RNase A treatment, diluted in PBS for 30 minutes at 40°C prior to probe hybridisation, to ensure the targeting of HSV1 DNA (**S1B Fig**). No probe controls in which no target probes were added also confirmed the specificity of the probe signal. Slides were then subject to six rounds of amplification according to the manufacturer’s protocol, followed by a 1.5 minute development step with FastRed A+B substrate solution. Sections were labelled with primary antibodies for 1 hour at room temperature or overnight at 4°C in the dark. Primary antibodies used for tissue sections include rabbit anti-GFP (polyclonal, Abcam, UK), goat anti-human langerin (polyclonal, R&D Systems, USA), mouse anti-human CD11c (3.9, eBioscience, USA), mouse anti-human nectin-1 (R1.302, BioLegend, USA) and mouse anti-human plakoglobin (5.1, Progen, Germany). Mouse nectin-1 was conjugated in house to Cyanine5 using the sulfo-Cyanine5 antibody conjugation kit (Lumiprobe, USA) to avoid cross-bonding of antibodies of the same species. Slides were washed twice in TBS for 5 minutes total and subject to secondary antibody staining for 30 minutes at room temperature in the dark. Secondary antibodies used include Alexa Fluor (AF)-488 conjugated donkey anti-rabbit IgG, AF-555 conjugated donkey anti-rabbit IgG, AF-546 conjugated donkey anti-mouse IgG, AF-647 conjugated donkey anti-goat IgG and donkey anti-mouse IgG, AF-755 conjugated donkey anti-goat IgG and donkey anti-mouse IgG (Life Technologies). Slides were washed as above, followed by 4′,6-diamidino-2-phenylindole (DAPI) nuclear stain at 1:1000 for 3 minutes, mounted with 20 x 24 mm rectangle coverslips (Menzel-Gläser, Germany) using SlowFade Diamond Antifade (Molecular Probes, USA), and sealed with nail polish. To detect epidermal MNPs with RNAscope, tissue was first labelled for CD11c, Langerin and GFP and imaged, prior to RNAscope as the process appeared to cleave or quench CD11c signal. The slides were stained, mounted but not sealed, and imaged as per usual, and then submerged in PBS for at least 30 minutes-1 hour on a rocker, and the coverslips were gently removed by hand. Slides were washed and RNAscope was performed. The slides were re-stained for DAPI, coverslipped and sealed then re-imaged. The images were then aligned using the two DAPI stains and merged in FIJI using the Image Registration plugin.

### HSV infection and immunofluorescence staining of the HaCaT cell line

HaCaT cells grown to 80–100% confluency on coverslips within a 24-well plate were infected with HSV1-GFP or UV-inactivated HSV1-GFP at an MOI of 0.1. In the case of Foscarnet treatment, Foscarnet was added to the culture at 100 μg/mL in DMEM for 1 hour prior to addition of HSV1 and remained for the duration of the culture. Post infection, cells were washed in PBS and fixed in 4% PFA for 30 minutes at room temperature and transferred to a humidified chamber for immunofluorescence labelling. Coverslips, 13 mm in diameter, were labelled with primary antibodies for 1 hour at room temperature or overnight at 4°C, washed 3 times by pipetting 100 μL PBS to each coverslip and aspirating, before staining with secondary antibodies for 30 minutes at room temperature in the dark. Antibodies used include mouse anti-ICP27 (0119, Virostat, USA), rabbit anti-glycoprotein D (polyclonal, Abcam, USA), mouse anti-human nectin-1 (R1.302, BioLegend, USA), AF-488 conjugated donkey anti-rabbit IgG, and AF-546 conjugated donkey anti-mouse IgG. Secondary staining was followed by DAPI staining for 1.5 minutes before coverslips were washed and mounted onto slides with Prolong Diamond Antifade Reagent (Molecular Probes, USA). Slides were imaged at 40x and analysed using FIJI software.

### Cytokine treatment of HaCaT cell line

HaCaT cells grown to 80–100% confluency on coverslips within a 24-well plate were treated with cytokines and chemokines in combination or individually for 6 hours. Cytokines were added in serial dilutions of 10, 50, 100 pg/mL diluted in serum-free DMEM (Lonza), and included IL-6, IL-8, CCL2, CCL3, CCL4, CCL5, CXCL1, CXCL5, CXCL9, CXCL10, CXCL11. Mock conditions were cultured in serum-free DMEM only. After the 6-hour culture, cells were fixed in 4% PFA for 30 minutes, followed by immunofluorescence labelling of nectin-1 as previously described.

### LEGENDplex assay

Supernatants from HaCaT cells infected with HSV1-GFP (MOI 1) or DMEM as a mock control for either 12 or 24 hours were filtered using 100 kiloDalton (kDa) Amicon filters (Merck) by centrifugation for 30 minutes at 4000 g and stored at -80°C until use. Supernatants from 24h HSV1-GFP^+^ (1x10^8^ PFU/mL) inner foreskin explants were collected in Eppendorf tubes and frozen at -80°C. The LEGENDplex assay was performed by the Flow Cytometry Core Facility at the Westmead Scientific Platforms (Westmead, Australia) according to the manufacturer’s protocol with reagents supplied with the LEGENDplex Multiplex Inflammatory Panel 1, Proinflammatory Chemokine Panel Assay or the Human Essential Immune Response kits (BioLegend). Briefly, samples were diluted in buffer in a 96-well plate and incubated with capture beads for 2 hours, followed by detection antibodies for 1 hour at room temperature with constant shaking. A tertiary reagent, Streptavidin-PE, was added to the samples for a further 30 minutes under the same conditions. Samples were acquired using the BD LSRFortessa with FACSDiva (version 6.1.3) and analysed using the LEGENDplex data analysis suite (BioLegend, QOGNIT).

### Reverse transcriptase-quantitative PCR

0.5x10^6^ cells were either mock-treated or treated with 10 pg/mL of IL-8 or CCL3 for 2 or 6 hours. Total cellular RNA was extracted from the cells using the ISOLATE II RNA Mini Kit (Bioline) according to the manufacturer’s protocol. cDNA was synthesised using the AffinityScript qPCR cDNA Synthesis Kit (Agilent Technologies) according to the manufacturer’s protocol. In brief, 6 μl of the extracted RNA was converted into cDNA using AffinityScript Reverse Transcriptase and random hexamers with a single cycle of annealing, extension, and termination at 25°C for 5 minutes, 42°C for 20 minutes and 95°C for 5 minutes respectively.

Quantitative Real Time (q)PCR was performed on cDNA samples using the Brilliant II SYBR Kit (Agilent Technologies) according to the manufacturer’s protocol, with GAPDH used as a housekeeping gene. cDNA samples were diluted 1:10 in DNase-free H_2_O. Standards were ten-fold serially diluted. qPCR reactions were carried in a 25 μl volume containing 5 μl cDNA, 12.5 μl of the Brilliant II SYBR mix, H_2_0 and a mix of forward and reverse primers (300 nM) of either GAPDH (accession number: NM_002046 F primer sequence 5’-GAG TCA ACG GAT TTG GTC GT; R primer sequence 5’-GAC AAG CTT CCC GTT CTC AG) or nectin-1 (accession number: NM_002855 F primer sequence 5’-GGA AAG CCT CAC TCT CAA CG; R primer sequence 5’-TGA TGG GTC CCT TGA AGA AG). The reaction was amplified through a hot start activation (95°C for 10 minutes) followed by 40 cycles of denaturation (95°C for 30 seconds) and annealing/extension (60°C for 45 seconds) steps. Relative fluorescence units were recorded after each cycle using the CFX96 Opus Real-Time PCR Detection System (Bio-Rad) and analysed using the CFX Maestro Software (Bio-Rad). Standard curves for GAPDH and nectin-1 were generated and used to calculate relative nectin-1 expression, normalised to GAPDH expression. The fold change in nectin-1 expression in each cytokine treatment relative to mock was also calculated.

### Statistical analyses

Statistical analyses were performed using GraphPad Prism 10 software (version 10.0.3). Data was displayed as mean ± standard deviation (S.D.) unless stated otherwise. Due to variability between samples, unpaired parametric t-tests which assumed Gaussian distribution but with unequal variances using Welch’s correction were used to compare a variable between conditions or cell subsets (**Figs 2F, 4B and 5B**). Paired t tests were used to generate statistics for sample-matched pairs where Gaussian distribution was assumed (**Fig 3D and 3E**). One-way (**Fig 5A and 5B**) and two-way (**Fig 5D**) ANOVA tests with Dunnett’s multiple comparisons tests were used for multiple comparisons between column values compared to a control column and Tukey’s multiple comparisons was used to compare values in all columns (**Fig 4E**). The test and number of replicates (n) used for each dataset is included in the figure legend. Significance was displayed as *p<0.05, **p<0.01, ***p<0.001, ****p<0.0001.

## Supporting information

S1 Fig HSV1 UL30 RNAscope probe is specific for HSV1 DNA in inner foreskin epidermis. **(A)** Cryosectioned 24-hour mock or HSV1 (1x10^8^ PFU/mL) infected inner foreskin tissue was labelled by RNAscope to detect HSV1 (UL30) DNA or PPIB positive control probe (red) and DAPI nuclear stain (grey). **(B)** HSV1 DNA probe was validated to confirm specificity of DNA targeting. Foreskin sections were treated with either RNase (100 μg/mL) or increasing concentrations of DNase for 30 minutes at 40°C prior to RNAscope detection of HSV1 DNA. Scale bars = 100 μm.
(TIF)

S2 Fig Vaxxas HD-MAPs puncture beyond the basement membrane of infant inner foreskin epidermis. **(A)** Vaxxas HD-MAPs were applied to the inner foreskin epidermis of adult (17+ y.o.), child (5–14 y.o.) and infant (< 5 y.o.) samples, immediately fixed in 4% PFA for 24 hours and paraffin-embedded. Tissue sections were stained for Haematoxylin and Eosin and images were acquired. Dotted line indicates the basement membrane (BM). Scale bars = 100 μm. **(B) (i)** The average thickness of each epidermis (μm), **(ii)** depth of punctures (μm), **(iii)** puncture depth as a proportion (%) of the total epidermal thickness, **(iv)** frequency of punctures per mm and **(v)** proportion (%) of punctures that reached the BM in multiple samples for each age group displayed as mean ± S.D. (adult: n = 4; 17, 21, 41 & 43 y.o., child: n = 5; 5, 7, 9, 12 & 14 y.o., infant: n = 2; 6 & 8 month old). ** = p<0.01, determined by unpaired parametric t tests with Welch’s correction assuming unequal variances.
(TIF)

S3 Fig HSV1 DNA probe detection in inner foreskin epidermis in the presence or absence of DNase. **(A)** Human inner foreskin infected with HSV1-GFP for 24h via HD-MAP was incubated with PBS (mock) or DNase (200 μg/mL) for 30mins at 40°C in the HybEZ hybridisation oven. Slides were labelled by RNAscope to detect HSV1 DNA (red), anti-GFP primary (green) and and DAPI nuclear stain (grey). Images were acquired on the Olympus VS200 Slide Scanner at 20x magnification. Scale bars = 100 μm.
(TIF)

S4 Fig Local migration of LCs and Epi DCs is observed 24 hours after induction of microtrauma. **(A-C)** Inner foreskin with or without Vaxxas HD-MAP treatment was either snap-frozen immediately (0 h) or after culture for 24 hours. Cryosectioned samples were labelled with anti-Langerin (blue) and anti-CD11c (red) antibodies and DAPI nuclear stain (grey) and imaged. **(A)** The density (cells/mm^2^) of **(i)** LCs and **(ii)** Epi DCs with and without patch treatment displayed as mean ± S.D. where each dot represents a sample. **(B)** Time course of the density (cells/mm^2^) of LCs and Epi DCs after patch-treatment in a single foreskin sample plotted as mean ± S.D. where each dot represents a section of tissue. **(C)** Representative image of langerin^+^CD11c^-^ LCs (green arrows) and Langerin^+/-^CD11c^+^ Epi DCs (cyan arrow) in inner foreskin cultured for 24 hours after Vaxxas HD-MAP treatment, located away from the puncture region (dotted yellow line). Scale bar = 50 μm.
(TIF)

S5 Fig Nectin-1 staining increases in keratinocytes surrounding HSV1 foci, but decreases within the foci. HaCaT cells grown on coverslips were treated with serum-free media for 24 hours or infected with HSV1-GFP at a MOI of 0.1 at timepoints of 6, 12 and 24 hours, after which cells were fixed in 4% PFA and labelled with anti-ICP27 and anti-Nectin-1 antibodies. Yellow arrows indicate nectin-1^+^ICP27^+^GFP^+^ cells, white arrows indicate nectin-1^+^ICP27^-^GFP^-^ cells. Scale bar = 100 μm.
(TIF)

S6 Fig Chemokine production in supernatants of HSV1^+^ inner foreskin explants. **(A)** Vaxxas HD-MAPs were pre-treated with HSV1-GFP (1x10^8^ pfu/mL) or media only (mock) and then applied to inner foreskin tissue. Tissue was cultured for 24 hours at 37°C. The supernatants were collected and analysed via the LEGENDplex Assay (BioLegend). Concentrations (pg/mL) of detected cytokines and chemokines are displayed where each paired dot represents a sample and lines connect sample-matched pairs (IL-6: n = 2, all others n = 3).
(TIF)

S7 Fig Cycling of Nectin-1 in HaCaT cell culture over time. **(A–C)** HaCaT cells were cultured in DMEM + 10% FBS at seeding densities of 50 000, 75 000 and 100 000 cells for 12, 18, 24 or 36 hours, and labelled with anti-Nectin-1 (magenta) antibody and DAPI nuclear stain (grey) and imaged. **(A and C)** Representative images of **(A)** nectin-1 at each timepoint at a seeding density of 100 000 cells and **(C)** Distinct localization of nectin-1 within different cells; punctate staining within cell cytoplasm, diffuse staining in cell membrane are displayed. Scale bars = 100 μm unless otherwise indicated. **(B)(i)** The area of nectin-1 staining intensity and **(ii)** the proportion of nectin-1^+^ area of total area at different seeding densities and timepoints displayed as mean ± S.D.. ** = p<0.01, *** = p<0.001 determined by ordinary two-way ANOVA with Tukey’s multiple comparisons test with a single pooled variance.
(TIF)

S1 Data Source data used to generate all graphs in this article. File containing source data for all graphs generated in the article. Each tab displays the raw data for a separate figure, with data for each individual graph presented as tables.
(XLSX)

## References

[1] Ayoub HH, Chemaitelly H, Abu-Raddad LJ. Characterizing the transitioning epidemiology of herpes simplex virus type 1 in the USA: model-based predictions. BMC Med. 2019;17(1):57. doi: 10.1186/s12916-019-1285-x 30853029 PMC6410528

[2] Cunningham AL, Turner RR, Miller AC, Para MF, Merigan TC. Evolution of recurrent herpes simplex lesions. An immunohistologic study. J Clin Invest. 1985;75(1):226–33. doi: 10.1172/JCI111678 3880773 PMC423430

[3] Kim M, Truong NR, James V, Bosnjak L, Sandgren KJ, Harman AN, et al. Relay of herpes simplex virus between Langerhans cells and dermal dendritic cells in human skin. PLoS Pathog. 2015;11(4):e1004812. doi: 10.1371/journal.ppat.1004812 25875649 PMC4395118

[4] Petermann P, Thier K, Rahn E, Rixon FJ, Bloch W, Ozcelik S, et al. Entry mechanisms of herpes simplex virus 1 into murine epidermis: involvement of nectin-1 and herpesvirus entry mediator as cellular receptors. J Virol. 2015;89(1):262–74. doi: 10.1128/JVI.02917-14 25320325 PMC4301110

[5] De La Cruz N, Möckel M, Wirtz L, Sunaoglu K, Malter W, Zinser M, et al. Ex vivo infection of human skin with herpes simplex virus 1 reveals mechanical wounds as insufficient entry portals via the skin surface. J Virol. 2021. doi: 10.1128/JVI.01338-21 34379501 PMC8513464

[6] Baggaley RF, White RG, Boily MC. HIV transmission risk through anal intercourse: systematic review, meta-analysis and implications for HIV prevention. Int J Epidemiol. 2010;39(4):1048–63. doi: 10.1093/ije/dyq057 20406794 PMC2929353

[7] Brawner BM, Sommers MS, Moore K, Aka-James R, Zink T, Brown KM, et al. Exploring Genitoanal Injury and HIV Risk Among Women: Menstrual Phase, Hormonal Birth Control, and Injury Frequency and Prevalence. J Acquir Immune Defic Syndr. 2016;71(2):207–12. doi: 10.1097/QAI.0000000000000824 26334741 PMC4712081

[8] Zink T, Fargo JD, Baker RB, Buschur C, Fisher BS, Sommers MS. Comparison of methods for identifying ano-genital injury after consensual intercourse. J Emerg Med. 2010;39(1):113–8. doi: 10.1016/j.jemermed.2008.08.024 19217245 PMC2917333

[9] Song SH, Fernandes JR. Comparison of Injury Patterns in Consensual and Nonconsensual Sex: Is It Possible to Determine if Consent was Given? Acad Forensic Pathol. 2017;7(4):619–31. doi: 10.23907/2017.052 31240011 PMC6474446

[10] Mehta SD, Krieger JN, Agot K, Moses S, Ndinya-Achola JO, Parker C, et al. Circumcision and reduced risk of self-reported penile coital injuries: results from a randomized controlled trial in Kisumu, Kenya. J Urol. 2010;184(1):203–9. doi: 10.1016/j.juro.2010.03.015 20483156 PMC3090633

[11] Westercamp N, Mehta SD, Jaoko W, Okeyo TA, Bailey RC. Penile coital injuries in men decline after circumcision: Results from a prospective study of recently circumcised and uncircumcised men in western Kenya. PLoS One. 2017;12(10):e0185917. doi: 10.1371/journal.pone.0185917 29016638 PMC5634596

[12] Schiffer JT, Abu-Raddad L, Mark KE, Zhu J, Selke S, Magaret A, et al. Frequent release of low amounts of herpes simplex virus from neurons: results of a mathematical model. Sci Transl Med. 2009;1(7):7ra16. doi: 10.1126/scitranslmed.3000193 20161655 PMC2818652

[13] Schiffer JT, Abu-Raddad L, Mark KE, Zhu J, Selke S, Koelle DM, et al. Mucosal host immune response predicts the severity and duration of herpes simplex virus-2 genital tract shedding episodes. Proc Natl Acad Sci U S A. 2010;107(44):18973–8. doi: 10.1073/pnas.1006614107 20956313 PMC2973882

[14] Bertram KM, Truong NR, Smith JB, Kim M, Sandgren KJ, Feng KL, et al. Herpes Simplex Virus type 1 infects Langerhans cells and the novel epidermal dendritic cell, Epi-cDC2s, via different entry pathways. PLoS Pathog. 2021;17(4):e1009536. doi: 10.1371/journal.ppat.1009536 33905459 PMC8104422

[15] Madavaraju K, Koganti R, Volety I, Yadavalli T, Shukla D. Herpes Simplex Virus Cell Entry Mechanisms: An Update. Front Cell Infect Microbiol. 2020;10:617578. doi: 10.3389/fcimb.2020.617578 33537244 PMC7848091

[16] Sayers CL, Elliott G. Herpes Simplex Virus 1 Enters Human Keratinocytes by a Nectin-1-Dependent, Rapid Plasma Membrane Fusion Pathway That Functions at Low Temperature. J Virol. 2016;90(22):10379–89. doi: 10.1128/JVI.01582-16 27630229 PMC5105671

[17] Basler K, Bergmann S, Heisig M, Naegel A, Zorn-Kruppa M, Brandner JM. The role of tight junctions in skin barrier function and dermal absorption. J Control Release. 2016;242:105–18. doi: 10.1016/j.jconrel.2016.08.007 27521894

[18] Brandner JM, Zorn-Kruppa M, Yoshida T, Moll I, Beck LA, De Benedetto A. Epidermal tight junctions in health and disease. Tissue barriers. 2015;3(e974451):1–2. doi: 10.4161/21688370.2014.974451 25838981 PMC4372028

[19] Harris TJ, Tepass U. Adherens junctions: from molecules to morphogenesis. Nat Rev Mol Cell Biol. 2010;11(7):502–14. doi: 10.1038/nrm2927 20571587

[20] Lewis JE, Wahl J. K. 3rd, Sass K. M., Jensen P. J., Johnson K. R., & Wheelock M. J. Cross-talk between adherens junctions and desmosomes depends on plakoglobin. The Journal of cell biology. 1997;136(4):919–34. doi: 10.1083/jcb.136.4.919 9049256 PMC2132504

[21] Kartenbeck J, Schmelz M, Franke WW, Geiger B. Endocytosis of junctional cadherins in bovine kidney epithelial (MDBK) cells cultured in low Ca2+ ion medium. J Cell Biol. 1991;113(4):881–92. doi: 10.1083/jcb.113.4.881 2026652 PMC2288996

[22] Asakura T, Nakanishi H, Sakisaka T, Takahashi K, Mandai K, Nishimura M, et al. Similar and differential behaviour between the nectin-afadin-ponsin and cadherin-catenin systems during the formation and disruption of the polarized junctional alignment in epithelial cells. Genes Cells. 1999;4(10):573–81. doi: 10.1046/j.1365-2443.1999.00283.x 10583506

[23] Yoon M, Spear PG. Disruption of adherens junctions liberates nectin-1 to serve as receptor for herpes simplex virus and pseudorabies virus entry. J Virol. 2002;76(14):7203–8. doi: 10.1128/jvi.76.14.7203-7208.2002 12072519 PMC136315

[24] Rahn E, Thier K, Petermann P, Rubsam M, Staeheli P, Iden S, et al. Epithelial Barriers in Murine Skin during Herpes Simplex Virus 1 Infection: The Role of Tight Junction Formation. J Invest Dermatol. 2017;137(4):884–93. doi: 10.1016/j.jid.2016.11.027 27939379

[25] Thier K, Petermann P, Rahn E, Rothamel D, Bloch W, Knebel-Morsdorf D. Mechanical Barriers Restrict Invasion of Herpes Simplex Virus 1 into Human Oral Mucosa. J Virol. 2017;91(22). doi: 10.1128/JVI.01295-17 28878080 PMC5660485

[26] Blaskewicz CD, Pudney J, Anderson DJ. Structure and function of intercellular junctions in human cervical and vaginal mucosal epithelia. Biol Reprod. 2011;85(1):97–104. doi: 10.1095/biolreprod.110.090423 21471299 PMC3123383

[27] Lemos MP, Lama JR, Karuna ST, Fong Y, Montano SM, Ganoza C, et al. The inner foreskin of healthy males at risk of HIV infection harbors epithelial CD4+ CCR5+ cells and has features of an inflamed epidermal barrier. PLoS One. 2014;9(9):e108954. doi: 10.1371/journal.pone.0108954 25268493 PMC4182607

[28] Ganor Y, Bomsel M. HIV-1 transmission in the male genital tract. Am J Reprod Immunol. 2011;65(3):284–91. doi: 10.1111/j.1600-0897.2010.00933.x 21114566

[29] Iwasaki A. Antiviral immune responses in the genital tract: clues for vaccines. Nat Rev Immunol. 2010;10(10):699–711. doi: 10.1038/nri2836 20829886 PMC3678359

[30] Bertram KM, Botting RA, Baharlou H, Rhodes JW, Rana H, Graham JD, et al. Identification of HIV transmitting CD11c(+) human epidermal dendritic cells. Nat Commun. 2019;10(1):2759. doi: 10.1038/s41467-019-10697-w 31227717 PMC6588576

[31] Cunningham AL, Carbone F, Geijtenbeek TB. Langerhans cells and viral immunity. Eur J Immunol. 2008;38(9):2377–85. doi: 10.1002/eji.200838521 18792031

[32] Stiles KM, Milne RS, Cohen GH, Eisenberg RJ, Krummenacher C. The herpes simplex virus receptor nectin-1 is down-regulated after trans-interaction with glycoprotein D. Virology. 2008;373(1):98–111. doi: 10.1016/j.virol.2007.11.012 18076965 PMC2629994

[33] Bhargava AK, Rothlauf PW, Krummenacher C. Herpes simplex virus glycoprotein D relocates nectin-1 from intercellular contacts. Virology. 2016;499:267–77. doi: 10.1016/j.virol.2016.09.019 27723487 PMC5172392

[34] Boelsma E, Verhoeven MCH, Ponec M. Reconstruction of a Human Skin Equivalent Using a Spontaneously Transformed Keratinocyte Cell Line (HaCaT). Journal of Investigative Dermatology. 1999;12(4):489–98. doi: 10.1046/j.1523-1747.1999.00545.x 10201534

[35] Colombo I, Sangiovanni E, Maggio R, Mattozzi C, Zava S, Corbett Y, et al. HaCaT Cells as a Reliable In Vitro Differentiation Model to Dissect the Inflammatory/Repair Response of Human Keratinocytes. Mediators of Inflammation. 2017;2017:1–12. doi: 10.1155/2017/7435621 29391667 PMC5748104

[36] Denes CE, Everett RD, Diefenbach RJ. Tour de Herpes: Cycling Through the Life and Biology of HSV-1. Methods Mol Biol. 2020;2060:1–30. doi: 10.1007/978-1-4939-9814-2_1 31617170

[37] Ibanez FJ, Farias MA, Gonzalez-Troncoso MP, Corrales N, Duarte LF, Retamal-Diaz A, et al. Experimental Dissection of the Lytic Replication Cycles of Herpes Simplex Viruses in vitro. Front Microbiol. 2018;9:2406. doi: 10.3389/fmicb.2018.02406 30386309 PMC6198116

[38] Oh MJ, Akhtar J, Desai P, Shukla D. A role for heparan sulfate in viral surfing. Biochem Biophys Res Commun. 2010;391(1):176–81. doi: 10.1016/j.bbrc.2009.11.027 19909728 PMC2812628

[39] Glass M, Everett RD. Components of promyelocytic leukemia nuclear bodies (ND10) act cooperatively to repress herpesvirus infection. J Virol. 2013;87(4):2174–85. doi: 10.1128/JVI.02950-12 23221561 PMC3571464

[40] Sekine E, Schmidt N, Gaboriau D, O’Hare P. Spatiotemporal dynamics of HSV genome nuclear entry and compaction state transitions using bioorthogonal chemistry and super-resolution microscopy. PLoS Pathog. 2017;13(11):e1006721. doi: 10.1371/journal.ppat.1006721 29121649 PMC5697887

[41] Zhang L-J. Keratins in Skin Epidermal Development and Diseases. In: IntechOpen, editor. Keratin: Miroslav Blumenberg; 2018.

[42] Jiang Y, Tsoi LC, Billi AC, Ward NL, Harms PW, Zeng C, et al. Cytokinocytes: the diverse contribution of keratinocytes to immune responses in skin. JCI Insight. 2020;5(20). doi: 10.1172/jci.insight.142067 33055429 PMC7605526

[43] Krummenacher C, Baribaud I, Sanzo JF, Cohen GH, Eisenberg RJ. Effects of herpes simplex virus on structure and function of nectin-1/HveC. J Virol. 2002;76(5):2424–33. doi: 10.1128/jvi.76.5.2424-2433.2002 11836420 PMC153823

[44] Huber MT, Wisner TW, Hegde NR, Goldsmith KA, Rauch DA, Roller RJ, et al. Herpes simplex virus with highly reduced gD levels can efficiently enter and spread between human keratinocytes. J Virol. 2001;75(21):10309–18. doi: 10.1128/JVI.75.21.10309-10318.2001 11581399 PMC114605

[45] Abaitua F, Zia FR, Hollinshead M, O’Hare P. Polarized cell migration during cell-to-cell transmission of herpes simplex virus in human skin keratinocytes. J Virol. 2013;87(14):7921–32. doi: 10.1128/JVI.01172-13 23658449 PMC3700176

[46] Boukamp P, Petrussevska RT, Breitkreutz D, Hornung J, Markham A, Fusenig NE. Normal keratinization in a spontaneously immortalized aneuploid human keratinocyte cell line. J Cell Biol. 1988;106(3):761–71. doi: 10.1083/jcb.106.3.761 2450098 PMC2115116

[47] Bragulla HH, Homberger DG. Structure and functions of keratin proteins in simple, stratified, keratinized and cornified epithelia. J Anat. 2009;214(4):516–59. doi: 10.1111/j.1469-7580.2009.01066.x 19422428 PMC2736122

[48] Mikloska Z, Danis VA, Adams S, Lloyd AR, Adrian DL, Cunningham AL. In vivo production of cytokines and beta (C-C) chemokines in human recurrent herpes simplex lesions—do herpes simplex virus-infected keratinocytes contribute to their production? J Infect Dis. 1998;177(4):827–38. doi: 10.1086/515236 9534953

[49] Thapa M, Carr DJ. Chemokines and Chemokine Receptors Critical to Host Resistance following Genital Herpes Simplex Virus Type 2 (HSV-2) Infection. Open Immunol J. 2008;1:33–41. doi: 10.2174/1874226200801010033 19043604 PMC2586324

[50] Zhang C, Li Z, Xu L, Che X, Wen T, Fan Y, et al. CXCL9/10/11, a regulator of PD-L1 expression in gastric cancer. BMC Cancer. 2018;18(1):462. doi: 10.1186/s12885-018-4384-8 29690901 PMC5916585

[51] Cheng Y, Ma XL, Wei YQ, Wei XW. Potential roles and targeted therapy of the CXCLs/CXCR2 axis in cancer and inflammatory diseases. Biochim Biophys Acta Rev Cancer. 2019;1871(2):289–312. doi: 10.1016/j.bbcan.2019.01.005 30703432

[52] Zhang C, Deng Y, Zhang Y, Ba T, Niu S, Chen Y, et al. CXCR3 Inhibition Blocks the NF-κB Signaling Pathway by Elevating Autophagy to Ameliorate Lipopolysaccharide-Induced Intestinal Dysfunction in Mice. Cells. 2023;12(1). 10.3390/cells12010182 PMC9818741 36611975

[53] Richmond A. Nf-kappa B, chemokine gene transcription and tumour growth. Nat Rev Immunol. 2002;2(9):664–74. doi: 10.1038/nri887 12209135 PMC2668257

[54] Jin WJ, Kim B, Kim D, Park Choo HY, Kim HH, Ha H, et al. NF-κB signaling regulates cell-autonomous regulation of CXCL10 in breast cancer 4T1 cells. Exp Mol Med. 2017;49(2):e295. 28209986 10.1038/emm.2016.148 PMC5336559

[55] Lee MM, Wong YH. CCR1-mediated activation of Nuclear Factor-kappaB in THP-1 monocytic cells involves Pertussis Toxin-insensitive Galpha(14) and Galpha(16) signaling cascades. J Leukoc Biol. 2009;86(6):1319–29. doi: 10.1189/jlb.0209052 19687291

[56] Čokić VP, Mitrović-Ajtić O, Beleslin-Čokić BB, Marković D, Buač M, Diklić M, et al. Proinflammatory Cytokine IL-6 and JAK-STAT Signaling Pathway in Myeloproliferative Neoplasms. Mediators Inflamm. 2015;2015:453020. doi: 10.1155/2015/453020 26491227 PMC4602333

[57] Sreenivasan L, Wang H, Yap SQ, Leclair P, Tam A, Lim CJ. Autocrine IL-6/STAT3 signaling aids development of acquired drug resistance in Group 3 medulloblastoma. Cell Death Dis. 2020;11(12):1035. doi: 10.1038/s41419-020-03241-y 33279931 PMC7719195

[58] Kaltschmidt C, Banz-Jansen C, Benhidjeb T, Beshay M, Förster C, Greiner J, et al. A Role for NF-κB in Organ Specific Cancer and Cancer Stem Cells. Cancers (Basel). 2019;11(5). 10.3390/cancers11050655 PMC6563002 31083587

[59] Wang L, Walia B, Evans J, Gewirtz AT, Merlin D, Sitaraman SV. IL-6 induces NF-kappa B activation in the intestinal epithelia. J Immunol. 2003;171(6):3194–201. doi: 10.4049/jimmunol.171.6.3194 12960348

[60] Zegeye MM, Lindkvist M, Fälker K, Kumawat AK, Paramel G, Grenegård M, et al. Activation of the JAK/STAT3 and PI3K/AKT pathways are crucial for IL-6 trans-signaling-mediated pro-inflammatory response in human vascular endothelial cells. Cell Commun Signal. 2018;16(1):55. doi: 10.1186/s12964-018-0268-4 30185178 PMC6125866

[61] Fan Y, Mao R, Yang J. NF-κB and STAT3 signaling pathways collaboratively link inflammation to cancer. Protein Cell. 2013;4(3):176–85. 23483479 10.1007/s13238-013-2084-3 PMC4875500

[62] Bai D, Ueno L, Vogt PK. Akt-mediated regulation of NFkappaB and the essentialness of NFkappaB for the oncogenicity of PI3K and Akt. Int J Cancer. 2009;125(12):2863–70. doi: 10.1002/ijc.24748 19609947 PMC2767458

[63] Levy L, Hill CS. Smad4 dependency defines two classes of transforming growth factor {beta} (TGF-{beta}) target genes and distinguishes TGF-{beta}-induced epithelial-mesenchymal transition from its antiproliferative and migratory responses. Mol Cell Biol. 2005;25(18):8108–25. doi: 10.1128/MCB.25.18.8108-8125.2005 16135802 PMC1234333

[64] Pedrazzi M, Nash B, Meucci O, Brandimarti R. Molecular features contributing to virus-independent intracellular localization and dynamic behavior of the herpesvirus transport protein US9. PLoS One. 2014;9(8):e104634. doi: 10.1371/journal.pone.0104634 25133647 PMC4136771

[65] Bollenbach L, Buske J, Mäder K, Garidel P. Poloxamer 188 as surfactant in biological formulations—An alternative for polysorbate 20/80? Int J Pharm. 2022;620:121706. doi: 10.1016/j.ijpharm.2022.121706 35367584

