# Supplementary figures and images for: Herpes simplex virus spreads rapidly in human foreskin, partly driven by chemokine-induced redistribution of Nectin-1 on keratinocytes
Source: PLoS Pathog. 2024 Jun 10;20(6):e1012267. doi: 10.1371/journal.ppat.1012267 (PMC11164381; doi:10.1371/journal.ppat.1012267)

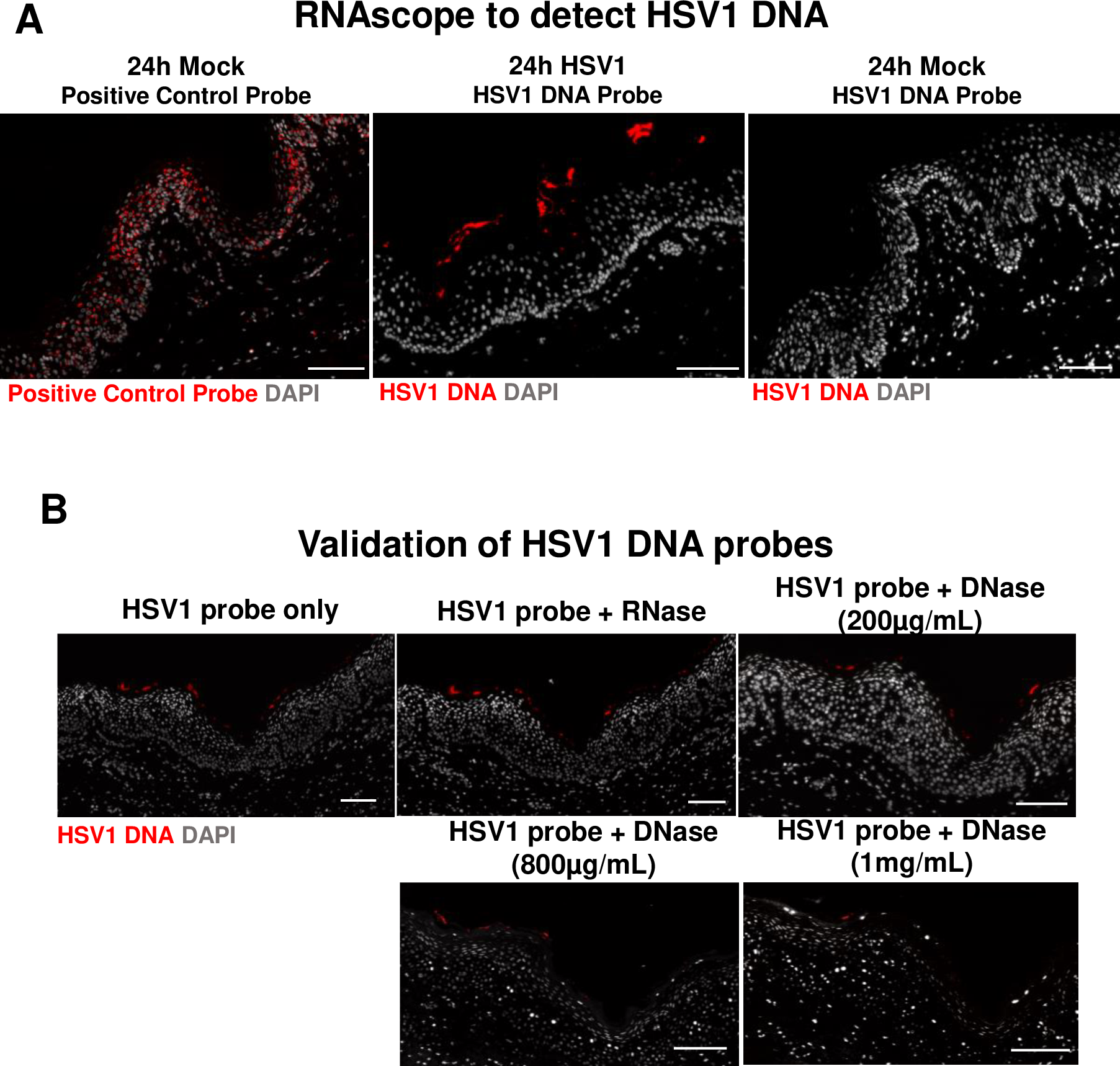

Supplement: S1 Fig — (A) Cryosectioned 24-hour mock or HSV1 (1x10^8^ PFU/mL) infected inner foreskin tissue was labelled by RNAscope to detect HSV1 (UL30) DNA or PPIB positive control probe (red) and DAPI nuclear stain (grey). (B) HSV1 DNA probe was validated to confirm specificity of DNA targeting. Foreskin sections were treated with either RNase (100 μg/mL) or increasing concentrations of DNase for 30 minutes at 40°C prior to RNAscope detection of HSV1 DNA. Scale bars = 100 μm. (TIF) [file ppat.1012267.s001.tif]

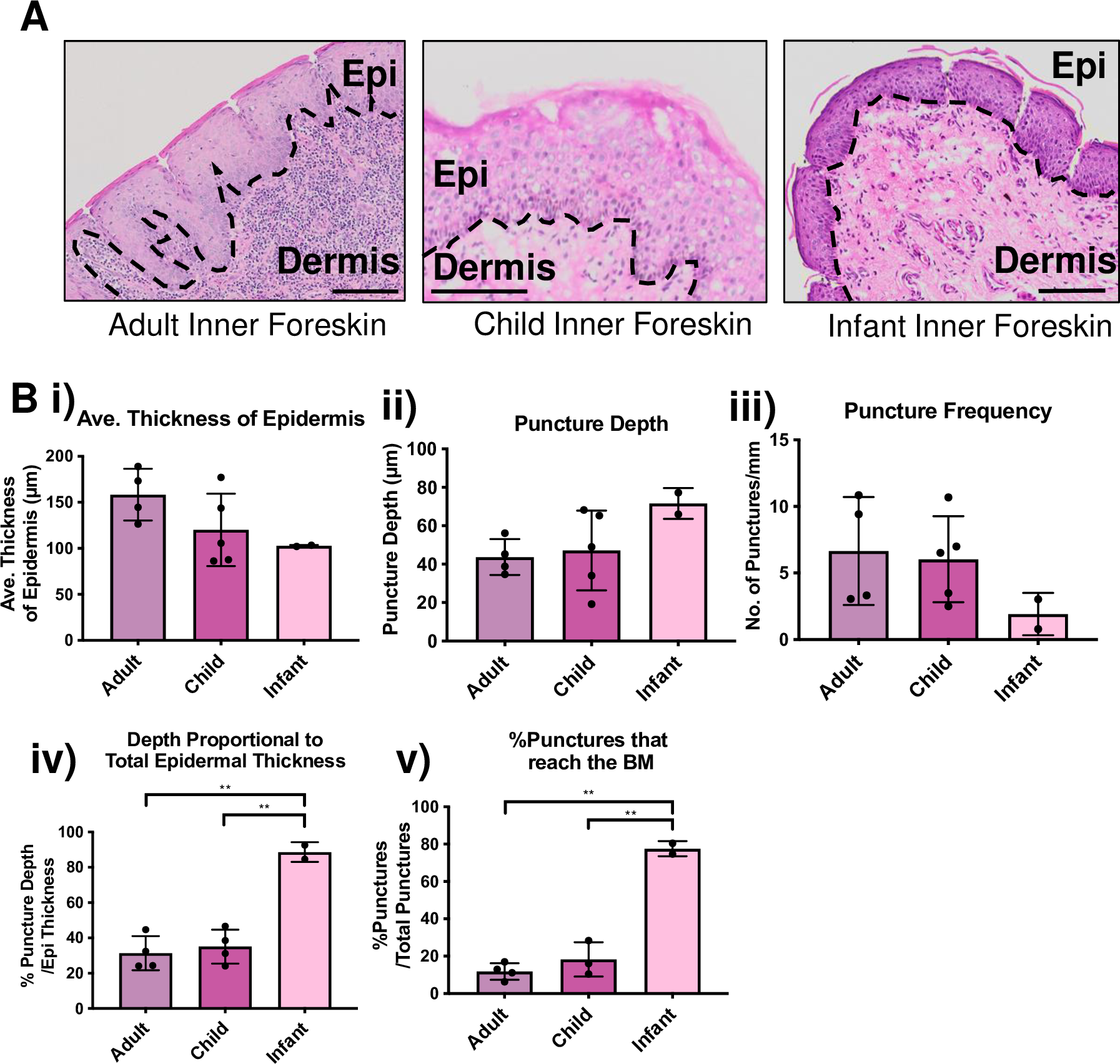

Supplement: S2 Fig — (A) Vaxxas HD-MAPs were applied to the inner foreskin epidermis of adult (17+ y.o.), child (5–14 y.o.) and infant (< 5 y.o.) samples, immediately fixed in 4% PFA for 24 hours and paraffin-embedded. Tissue sections were stained for Haematoxylin and Eosin and images were acquired. Dotted line indicates the basement membrane (BM). Scale bars = 100 μm. (B) (i) The average thickness of each epidermis (μm), (ii) depth of punctures (μm), (iii) puncture depth as a proportion (%) of the total epidermal thickness, (iv) frequency of punctures per mm and (v) proportion (%) of punctures that reached the BM in multiple samples for each age group displayed as mean ± S.D. (adult: n = 4; 17, 21, 41 & 43 y.o., child: n = 5; 5, 7, 9, 12 & 14 y.o., infant: n = 2; 6 & 8 month old). ** = p<0.01, determined by unpaired parametric t tests with Welch’s correction assuming unequal variances. (TIF) [file ppat.1012267.s002.tif]

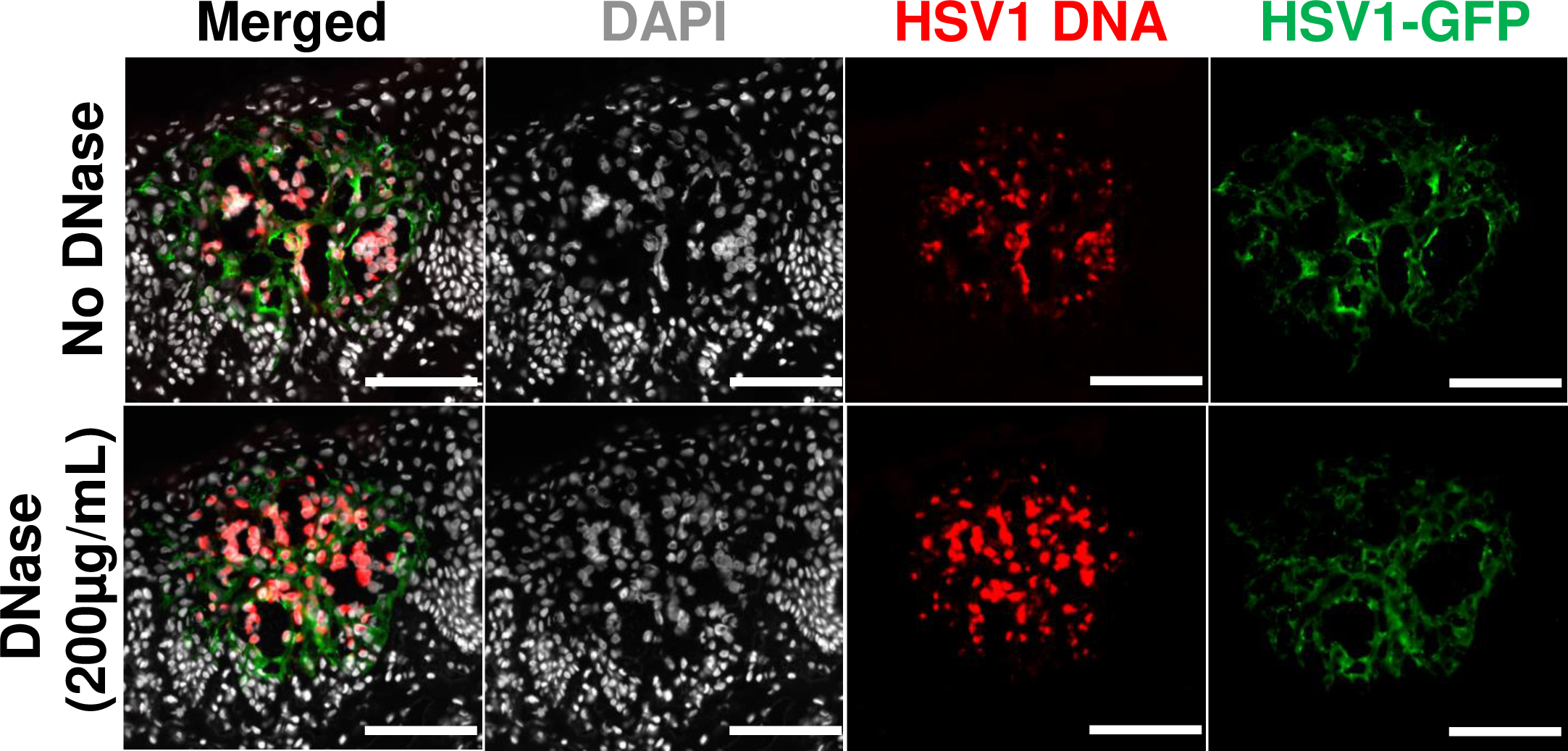

Supplement: S3 Fig — (A) Human inner foreskin infected with HSV1-GFP for 24h via HD-MAP was incubated with PBS (mock) or DNase (200 μg/mL) for 30mins at 40°C in the HybEZ hybridisation oven. Slides were labelled by RNAscope to detect HSV1 DNA (red), anti-GFP primary (green) and and DAPI nuclear stain (grey). Images were acquired on the Olympus VS200 Slide Scanner at 20x magnification. Scale bars = 100 μm. (TIF) [file ppat.1012267.s003.tif]

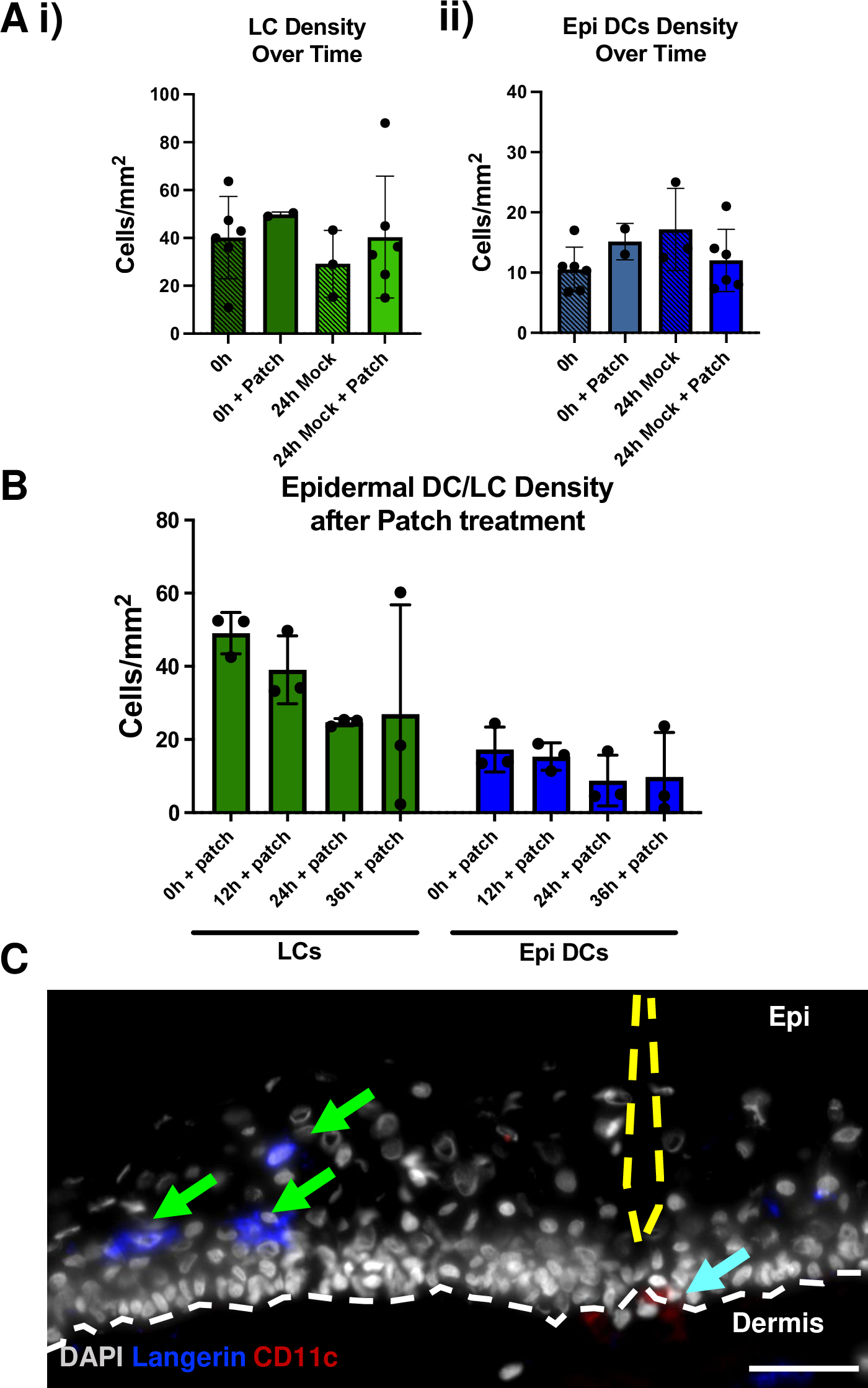

Supplement: S4 Fig — (A-C) Inner foreskin with or without Vaxxas HD-MAP treatment was either snap-frozen immediately (0 h) or after culture for 24 hours. Cryosectioned samples were labelled with anti-Langerin (blue) and anti-CD11c (red) antibodies and DAPI nuclear stain (grey) and imaged. (A) The density (cells/mm2) of (i) LCs and (ii) Epi DCs with and without patch treatment displayed as mean ± S.D. where each dot represents a sample. (B) Time course of the density (cells/mm2) of LCs and Epi DCs after patch-treatment in a single foreskin sample plotted as mean ± S.D. where each dot represents a section of tissue. (C) Representative image of langerin+CD11c- LCs (green arrows) and Langerin+/-CD11c+ Epi DCs (cyan arrow) in inner foreskin cultured for 24 hours after Vaxxas HD-MAP treatment, located away from the puncture region (dotted yellow line). Scale bar = 50 μm. (TIF) [file ppat.1012267.s004.tif]

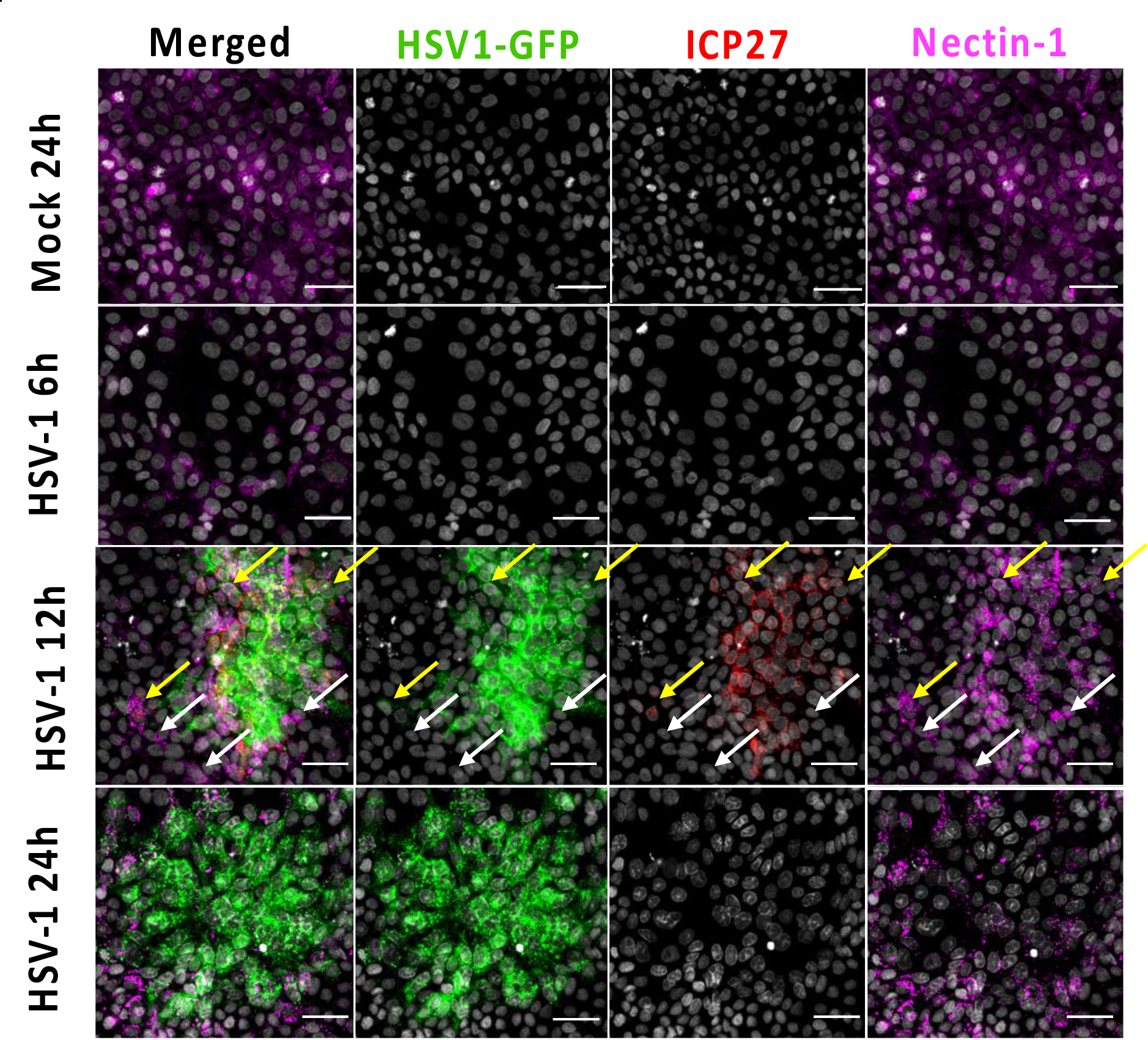

Supplement: S5 Fig — HaCaT cells grown on coverslips were treated with serum-free media for 24 hours or infected with HSV1-GFP at a MOI of 0.1 at timepoints of 6, 12 and 24 hours, after which cells were fixed in 4% PFA and labelled with anti-ICP27 and anti-Nectin-1 antibodies. Yellow arrows indicate nectin-1+ICP27+GFP+ cells, white arrows indicate nectin-1+ICP27-GFP- cells. Scale bar = 100 μm. (TIF) [file ppat.1012267.s005.tif]

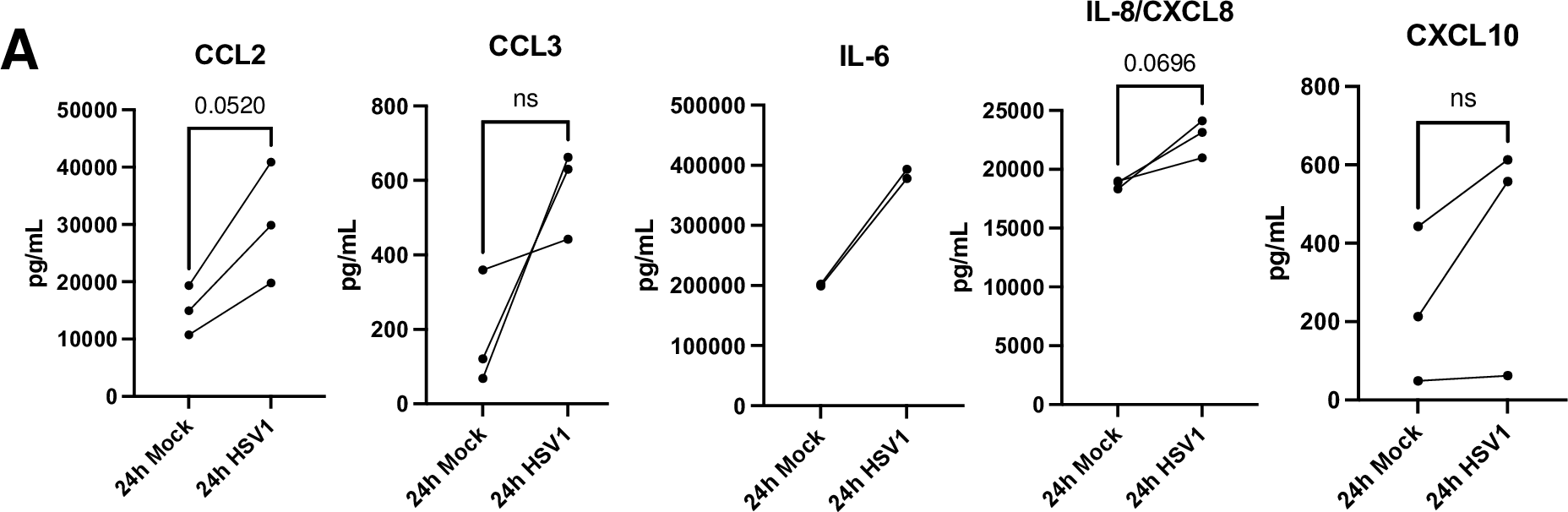

Supplement: S6 Fig — (A) Vaxxas HD-MAPs were pre-treated with HSV1-GFP (1x10^8^ pfu/mL) or media only (mock) and then applied to inner foreskin tissue. Tissue was cultured for 24 hours at 37°C. The supernatants were collected and analysed via the LEGENDplex Assay (BioLegend). Concentrations (pg/mL) of detected cytokines and chemokines are displayed where each paired dot represents a sample and lines connect sample-matched pairs (IL-6: n = 2, all others n = 3). (TIF) [file ppat.1012267.s006.tif]

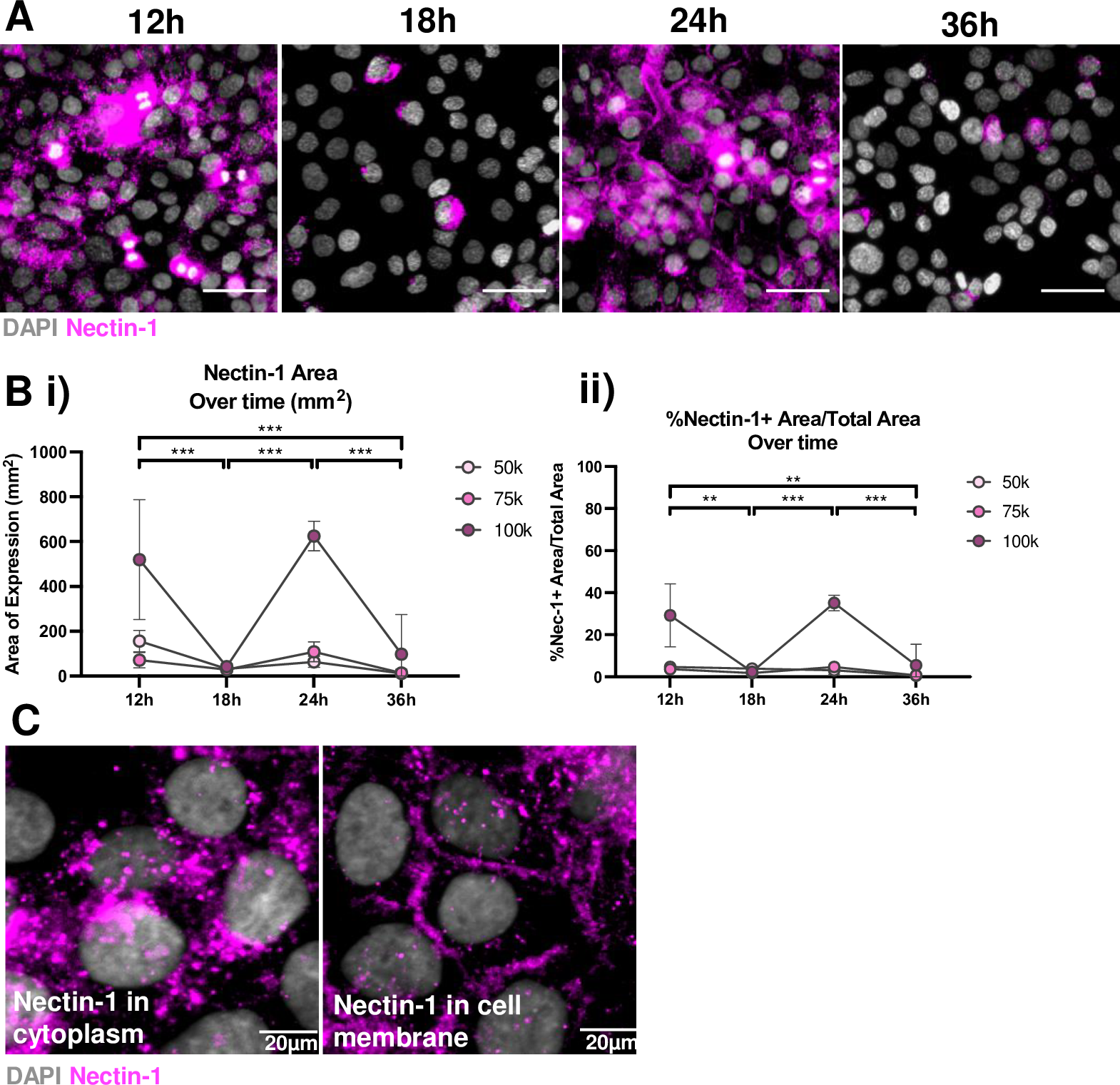

Supplement: S7 Fig — (A–C) HaCaT cells were cultured in DMEM + 10% FBS at seeding densities of 50 000, 75 000 and 100 000 cells for 12, 18, 24 or 36 hours, and labelled with anti-Nectin-1 (magenta) antibody and DAPI nuclear stain (grey) and imaged. (A and C) Representative images of (A) nectin-1 at each timepoint at a seeding density of 100 000 cells and (C) Distinct localization of nectin-1 within different cells; punctate staining within cell cytoplasm, diffuse staining in cell membrane are displayed. Scale bars = 100 μm unless otherwise indicated. (B)(i) The area of nectin-1 staining intensity and (ii) the proportion of nectin-1+ area of total area at different seeding densities and timepoints displayed as mean ± S.D.. ** = p<0.01, *** = p<0.001 determined by ordinary two-way ANOVA with Tukey’s multiple comparisons test with a single pooled variance. (TIF) [file ppat.1012267.s007.tif]
